# A Renal Clearable Probe for In Vivo Monoamine Oxidase (MAO) Detection

**DOI:** 10.1002/advs.202520797

**Published:** 2026-02-23

**Authors:** Marcia Domínguez, David Azorín‐Soriano, Araceli Lérida‐Viso, Jessie García‐Fleitas, Paula M Soriano‐Teruel, Jennifer Soler Beatty, Paula Rodrigo‐Martínez, Pau Arroyo, Sara Rojas‐Vázquez, Isabel Fariñas, Félix Sancenón, Juan F. Blandez, Alba García‐Fernández, Ramón Martínez‐Máñez

**Affiliations:** ^1^ Instituto Interuniversitario de Investigación de Reconocimiento Molecular y Desarrollo Tecnológico (IDM) Universitat Politècnica de València Universitat de València Valencia Spain; ^2^ CIBER de Bioingeniería, Biomateriales y Nanomedicina (CIBER‐BBN) Instituto de Salud Carlos III Madrid Spain; ^3^ Unidad Mixta UPV‐CIPF de Investigación en Mecanismos de Enfermedades y Nanomedicina, Universitat Politècnica de València Centro de Investigación Príncipe Felipe Valencia Spain; ^4^ Unidad Mixta de Investigación en Nanomedicina y Sensores Universitat Politècnica de València Avenida Fernando Abril Martorell Instituto de Investigación Sanitaria La Fe (IIS La Fe) Valencia Spain; ^5^ Instituto Interuniversitario de Investigación de Reconocimiento Molecular y Desarrollo Tecnológico Universitat de València, Universitat Politècnica de València Burjassot Valencia Spain; ^6^ Departamento de Química Orgánica Universitat de València Burjassot Valencia Spain; ^7^ Instituto de Biotecnología y Biomedicina (BIOTECMED) Universitat de València Valencia Spain; ^8^ CIBER de Enfermedades Neurodegenerativas (CIBERNED) Instituto de Salud Carlos III Madrid Spain; ^9^ Instituto de Ciencia Molecular (ICMol) Universidad de Valencia Paterna Valencia Spain

**Keywords:** monoamine oxidase, non‐invasive tool, renal clearable probe, zwitterionic compounds

## Abstract

Hyperactivation of monoamine oxidase enzymes (MAO) is associated with uncontrolled production of neurotoxic compounds such as reactive oxygen species, whose accumulation is linked to neurodegenerative, chronic, and age‐related diseases. Although chromo‐fluorogenic probes for detecting and quantifying MAO activity have been reported, non‐invasive methods for monitoring MAO overexpression in vivo remain elusive. Here, we report a renal‐clearable fluorogenic probe based on the cyanine‐7 fluorophore (**Cy7‐MAO**) for in vivo detection of MAO overexpression through a simple measurement of the fluorescence in urine. The probe incorporates sulfonic acid moieties for renal clearance and a propylamino group as a MAO substrate. Upon administration, **Cy7‐MAO** is hydrolysed at the site by MAO, releasing a highly emissive Cy7 fluorophore, which is excreted in urine and quantified by fluorescence. **Cy7‐MAO** is validated in vitro using HepG2 liver human cells, which express elevated MAO levels. We further demonstrate the in vivo applicability of **Cy7‐MAO** for MAO activity monitoring in aged mice, which show significantly higher urine fluorescence than young mice, consistent with an elevated MAO activity in older animals. These findings support **Cy7‐MAO** as a tool for longitudinal assessment of MAO activity in vivo, providing a novel approach to study MAO‐related pathologies.

## Graphical abstract

A renal‐clearable Cy7‐based fluorogenic probe enables rapid and non‐invasive detection of monoamine oxidase (MAO) activity in vivo. Upon MAO‐mediated activation, the fluorescent product is efficiently excreted in urine, allowing direct quantification of enzyme overexpression. This strategy provides a simple and longitudinal approach to monitor age‐associated MAO activity in living subjects.

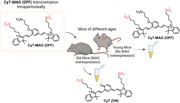

## 1 Introduction

Early and precise detection is crucial for effective diagnosis and treatment across a wide range of pathologies. Traditional diagnostic techniques, such as Magnetic Resonance Imaging (MRI), Positron Emission Tomography (PET), and Computed Tomography (CT), although widely used, have several limitations [1, 2, 3, 4]. These include high costs, the need for specialised equipment and trained personnel, and, in some cases, the administration of contrast agents that can cause adverse effects. Moreover, many other biomarker detection methods suffer from limited specificity and accumulation in tissues, increasing the risk of toxicity. In response to these challenges, the design of non‐invasive and cost‐effective diagnostic protocols is gaining significant interest. Urine‐based detection platforms represent a significant advancement, facilitating the identification of biomarkers and providing a robust complement or, in certain contexts, an alternative to conventional bioimaging modalities [5, 6].

A particularly innovative approach in the development of urine diagnostic tools is the preparation of probes that produce highly emissive fluorophores, which are subsequently excreted in urine. In this approach, an OFF‐state probe is transformed into a highly emissive ON‐state fluorophore by the action of a specific cellular biomarker. Moreover, the fluorophore is designed for rapid renal clearance, meaning it passes through the kidneys to reach the bladder for excretion in urine, enabling easy fluorescence detection in urine. Based on this mechanism, biomarkers can be detected via urinalysis, a technique that has been proven as a valuable tool for diagnosing and monitoring various medical conditions [7]. Recent studies have expanded on this concept, employing protease‐responsive nanoparticles [8, 9], fluorescent or chemiluminescent derivatives functionalized with β‐cyclodextrin (HPβCD) [10, 11], and other renal‐clearable organic molecular probes. Among the latter are hemicyanine‐based fluorophores conjugated to HPβCD or dextran backbones for detecting biomarkers such as Caspase‐3 [7], gamma‐glutamyl transferase [12], and SARS‐CoV‐2 [13]. In this context, we have also recently reported a probe that allows simple and semi‐quantitative assessment of senescence burden in vivo [5]. These examples represent the first reports of fluorogenic probes capable of detecting target biomarkers by urine analysis, highlighting the potential of renally activatable probes as accessible and clinically translatable diagnostic tools. Their ability to monitor biomarker dynamics longitudinally represents a distinct advantage for geriatric applications, where minimally invasive measurements are highly valuable. In addition, rapid elimination through urine minimizes tissue accumulation and potential toxicity. Encouraged by these promising results, we decided to extend this strategy to other clinically relevant biomarkers for which non‐invasive detection methods have not yet been fully developed.

Monoamine oxidase (MAO) enzymes represent a particularly compelling target due to their central role in neurotransmitter metabolism and the generation of hydrogen peroxide (H_2_O_2_) and other reactive oxygen species (ROS). These flavoenzymes are located on the outer mitochondrial membrane and can be found in most cell types in the body. MAO enzymes exist in two isoforms, MAO‐A and MAO‐B, which share a 70% sequence homology but different substrate and inhibitor specificities [14]. MAO‐A mainly cleaves serotonin, melatonin, noradrenaline, and adrenaline, while MAO‐B acts on phenethylamine and benzylamine [15]. During the oxidative deamination of these substrates, MAO activity produces H_2_O_2_ and other ROS as byproducts [16, 17, 18, 19]. Tissue‐specific changes in MAO levels have been described in age‐related disorders, as increased oxidative stress and mitochondrial dysfunction are key factors in cellular and organ function [20, 21]. For instance, the accumulation of ROS has been implicated in the pathogenesis of several neurodegenerative diseases, including Parkinson, Alzheimer or amyotrophic lateral sclerosis [22, 23, 24, 25], as well as chronic conditions such as endothelial dysfunction in hypertension, metabolic disorders, and chronic kidney disease [26, 27]. Many of these chronic conditions are highly prevalent in aging populations and contribute to the functional decline associated with aging [28].

Despite the involvement of MAO enzymes in various pathological and age‐related conditions, tools to monitor tissue‐specific changes in MAO in vivo are extremely limited or non‐existent. Several procedures have been described in the literature to detect MAO overexpression, such as Enzyme‐Linked Immunosorbent Assay (ELISA), High Performance Liquid Chromatography (HPLC), or spectrophotometric assays [29, 30, 31]. However, these approaches frequently rely on terminal procedures, such as tissue extraction or cell lysis, which preclude longitudinal measurements. As a result, their utility is largely confined to in vitro and ex vivo detection, with only limited instances successfully applied to in vivo monitoring (Table S1) [32, 33, 34]. Moreover, many probes rely on conventional fluorophores with poor aqueous solubility, low quantum yield in biological environments, or non‐specific background fluorescence and limited elimination. In this scenario, renal‐clearable OFF–ON fluorescent probes emerge as a promising alternative for in vivo dynamic monitoring of MAO activity.

**FIGURE 1:**
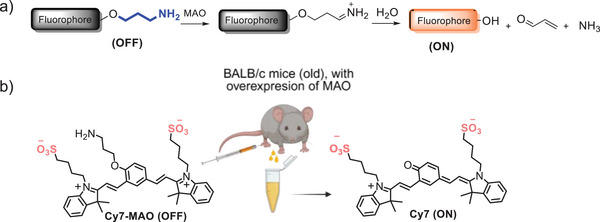
Design and performance of **Cy7‐MAO** probe. (a) Schematic illustration of the activatable OFF‐ON fluorescence probe for the detection of MAO enzymes. (b) Structure of the fluorescent probe **Cy7‐MAO** and the product Cy7 after catalytic oxidative deamination by MAO enzymes.

We report herein a fluorescent molecular probe, **Cy7‐MAO**, based on a cyanine‐7 fluorophore containing a propylamine group as recognition moiety (Figure 1a) [35, 36, 37, 38, 39, 40]. The sensing mechanism is based on an increase in fluorescence signal in the presence of MAO enzymes, which transform the weakly emissive **Cy7‐MAO** molecule into the high‐emissive Cy7 fluorophore. To achieve urinalysis, the probe backbone contains two sulfonic acid groups to enhance solubility and promote rapid renal clearance in vivo (Figure 1b), while the zwitterionic nature of the probe is also expected to reduce non‐specific protein binding and uptake by healthy tissues [5, 41, 42]. In this context, **Cy7‐MAO** provides a robust and versatile approach, enabling, for the first time, non‐invasive, longitudinal monitoring of MAO activity in living animals via urine collection. This design not only functions in vitro and ex vivo but also fills a critical gap in MAO research, providing unique biological insight into enzyme dynamics.

## 2 Results and Discussion

### 2.1 Synthesis and characterisation of Cy7‐MAO probe

**FIGURE 2:**
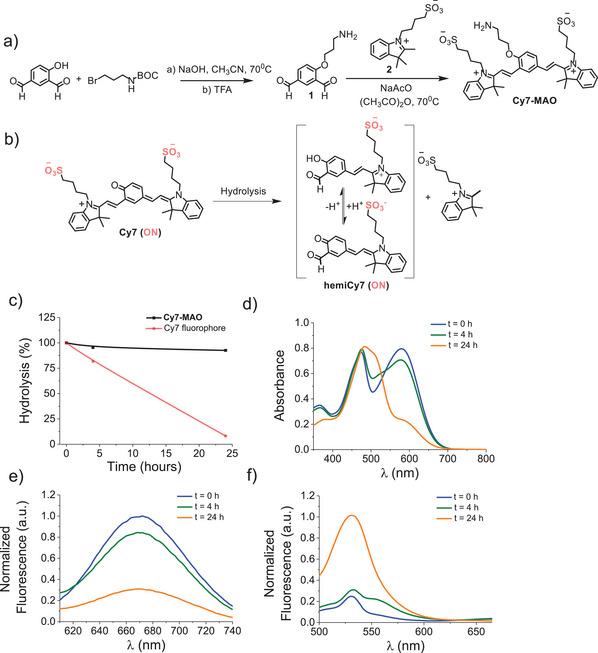
Synthesis and mechanistic studies of **Cy7‐MAO** hydrolysis. (a) Synthetic sequence to prepare **Cy7‐MAO**. (b) Hydrolysis of the Cy7 fluorophore. (c) % hydrolysis of **Cy7‐MAO** and Cy7 vs. time. The stability study was carried out by HPLC‐MS using an eluent gradient method from H_2_O‐acetonitrile (100:0 v/v) to H_2_O‐acetonitrile (0:100 v/v) at 20 min with a flow rate of 0.4 mL·min^−1^ with a kromasil C18 column. Mass spectroscopy chromatograms were recorded with an Agilent Ultivo mass spectrometer equipped with a triple Q‐TOF detector using a dual selected ion monitoring (SIM) function at 745 m/z and 705 m/z simultaneously, corresponding to the **Cy7‐MAO** and Cy7 fluorophore, respectively. (d) Absorption spectra of the Cy7 fluorophore (10^−4^ m in HEPES (10 mm, pH 7.4)) at different time points. (e), (f) Fluorescence emission spectra of the Cy7 fluorophore (10^−4^ m in HEPES (10 mm, pH 7.4)) at different time points, using excitation wavelengths at 580 nm (e) and 450 nm (f).

**Cy7‐MAO** was synthesized by a two‐step procedure (Figure 2a). In a first step, a nucleophilic substitution reaction between BOC‐protected 3‐bromopropylamine and 5‐formylsalycilaldehyde was carried out, and the BOC‐protecting group was removed with TFA, yielding the intermediate compound **1**. Then, in a second step, a Knoevenagel condensation between **1** and 2,3,3‐trimethyl‐1‐(4‐sulfobutyl) indolium (**2**) yielded the final **Cy7‐MAO** probe. **Cy7‐MAO** was purified by silica gel chromatography and fully characterized by ^1^H‐NMR, ^13^C‐NMR, and HRMS (Figures S1–S5). In addition, the Cy7 fluorophore was synthesized according to previously reported procedures (Figure S6) [5]. On the other hand, emission of Cy7 fluorophore solutions (20 µm in HEPES, 10 mm, pH 7.4) at different pH values corroborated fluorophore stability (in short periods of time) in the 5–10 pH range (Figure S7).

Cy7 fluorophore presents remarkable photophysical features for bioimaging (NIR emission), but its use in biological media requires careful evaluation as it can be hydrolyzed into a hemicyanine aldehyde (hemiCy7, see Figure 2b), which is also emissive [41]. In order to assess the applicability of the **Cy7‐MAO** probe, its stability together with that of Cy7 was tested through HPLC‐MS studies (Figure 2c; Figures S8 and S9). For this purpose, solutions of **Cy7‐MAO** and Cy7 (10^−4^ m in 10 mm HEPES, pH 7.4) were prepared, and mass spectrometry chromatograms were recorded using a mass spectrometer equipped with a triple Q‐TOF detector, which employed a dual selected ion monitoring (SIM) function at 745 m/z (**Cy7‐MAO** probe) and 705 m/z (Cy7 fluorophore) simultaneously. Mass spectra, at both m/z ratio, were recorded at different times, and the results obtained are depicted in Figure 2c. As could be seen, after 24 h, the abundance of the molecular ion selected to monitor the presence of the **Cy7‐MAO** (745 m/z corresponding to [M‐CH_3_] ^+^) remained unchanged, whereas that of the Cy7 fluorophore (705 m/z corresponding to [M+H] ^+^) drastically decreased (ca. 92%). This disappearance was associated with the hydrolysis of the Cy7 fluorophore to hemiCy7 (Figure 2b).

UV‐vis measurements also confirmed the proposed hydrolysis process (Figure 2d). HEPES solutions of Cy7 fluorophore showed a marked absorption band centered at ca. 600 nm which gradually decreased with time, while a new band appeared at ca. 450 nm, ascribed to hemiCy7 that was formed after Cy7 hydrolysis. The same results were obtained using fluorescence measurements (Figure 2e,f). Thus, upon excitation at 580 nm, the characteristic emission of Cy7 fluorophore at ca. 670 nm was observed (Figure 2e). However, this emission band was progressively reduced until it almost disappeared after 24 h. As a clear contrast, under excitation at 450 nm (absorption of hemiCy7), an emission band was found at ca. 535 nm after 24 h. These studies corroborate the formation of hemiCy7 after hydrolysis of Cy7. To corroborate these observations, hemiCy7 was also synthesized [43] and characterized by NMR, as well as by optical spectroscopic techniques, including UV‐vis absorption and fluorescence spectroscopy (Figures S10 and S11). These studies validate the proposed hydrolysis pathway and confirm the identity of the product resulting from the hydrolysis of Cy7. Despite the inherent instability of the Cy7 fluorophore at long time, the results also confirm that the **Cy7‐MAO** probe remains chemically stable in aqueous buffer at physiological pH. This stability supports its potential utility in biological applications, such as the detection of elevated MAO levels without degradation.

### 2.2 Selective MAO detection using the Cy7‐MAO probe

**FIGURE 3:**
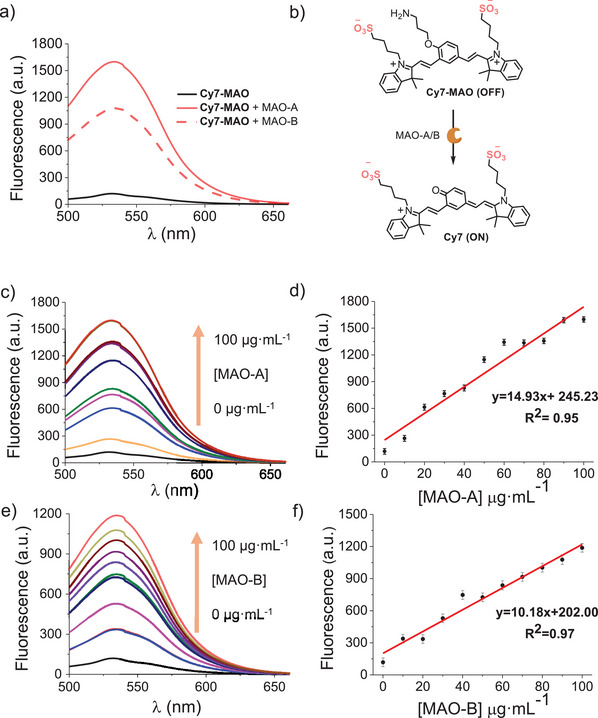
MAO‐mediated activation of **Cy7‐MAO**. (a) Fluorescence emission spectra (λ_exc_ = 450 nm) of **Cy7‐MAO** (5 µm) (black curve) and **Cy7‐MAO** (5 µm) + MAO‐A/B (100 µg·mL^−1^) (orange curves) in HEPES (10 mm, pH 7.4) after 24 h of incubation at 37°C. (b) MAO‐induced oxidative deamination of **Cy7‐MAO**. (c), (e) Fluorescence spectra of **Cy7‐MAO** (5 µm) in the presence of increasing MAO‐A/B concentrations (0–100 µg·mL^−1^) after 24 h of incubation at 37°C. (d), (f) Calibration curve of **Cy7‐MAO** for MAO‐A/B in HEPES solution (10 mm, pH 7.4). Error bars are expressed as 3σ from three independent experiments.

**Cy7‐MAO** was photophysically studied under simulated physiological conditions in the presence or absence of MAO‐A or MAO‐B enzymes. In a typical experiment, **Cy7‐MAO** (5 µm) in HEPES solution (10 mm, pH 7.4) was mixed with MAO‐A or MAO‐B (0–100 µg·mL^−1^) and the mixture was incubated at 37°C for 24 h to allow a complete enzyme‐mediated oxidative deamination of the probe. Emission after 24 h was measured at 535 nm (λ_ex_ = 450 nm) that is the emission of the hemiCy7 derivative obtained by hydrolysis of Cy7 after this time (*vide ante*). Starting **Cy7‐MAO** (5 µm) solutions in HEPES (10 mm, pH 7.4) were weakly emissive upon excitation at 450 nm, whereas, in sharp contrast (Figure 2a), a broad fluorescence band centered at 535 nm was observed upon addition of both enzymes (at 100 µg·mL^−1^) after 24 h (13.2‐ and 9.7‐fold emission enhancement for MAO‐A and MAO‐B, respectively). Next, the fluorescence emission of **Cy7‐MAO** probe (5 µm) was monitored after 24 h in the presence of different amounts of MAO‐A or MAO‐B (0–100 µg·mL^−1^) (Figure 3c,e). A significant increase in emission at 535 nm, proportional to the amount of enzyme added was found. These data were used to determine the LOD and LOQ for both enzymes (Figure 3d,f). LOD of 6.4 and 10.9 µg·mL^−1^ and LOQ values of 29.3 and 52.1 µg·mL^−1^ were found for MAO‐A and MAO‐B, respectively. These results indicate that the **Cy7‐MAO** probe can be used for the qualitative and quantitative detection of both MAO isoforms.

To further characterize its enzymatic behaviour, the kinetics of the reactions between **Cy7‐MAO** and MAO‐A and MAO‐B were investigated (Figure S12). The Michaelis–Menten analysis yielded the following catalytic parameters: for MAO‐A, a maximum velocity (V_max_) of 16.13 µm·min^−^^1^ and a Michaelis constant (K_m_) of 10.63 µm; for MAO‐B, a V_max_ of 35.71 µm·min^−^^1^ and a K_m_ of 17.82 µm. The lower K_m_ for MAO‐A indicates a higher apparent affinity of **Cy7‐MAO** for this isoform. In contrast, the higher V_max_ for MAO‐B reflects a greater catalytic capacity under substrate‐saturating conditions. These results demonstrate that **Cy7‐MAO** is efficiently recognised and processed by both MAO isoforms, albeit with distinct kinetic profiles, confirming its suitability as a sensitive and reliable probe for monitoring MAO activity. Taken together, these data provide strong experimental support for the use of **Cy7‐MAO** in enzymatic studies targeting both MAO‐A and MAO‐B.

To investigate and gain insights into the binding orientation and molecular interactions between **Cy7‐MAO** and the MAO‐A and MAO‐B isoforms, molecular docking studies (MD) were performed using the AutoDock Tools platform (MGL Tools suite v1.5.7). The results of the MD analysis showed that the ligand fits into the active site cavity of both isoforms. In some of the resulting clusters, the amino group‐containing end of **Cy7‐MAO** is seen to be close to the FAD cofactor, which is naturally present in MAO enzymes. The most promising complex of MAO‐A and MAO‐B with **Cy7‐MAO**, based on binding energy and more populated clusters, was subjected to stability analysis using MD simulations of 200 ns. The results of the simulations showed that the complexes with the two isoforms were stable during the simulation time, with the side chain containing the amino moiety remaining in the vicinity of FAD, which is stated as the catalytic site of the enzyme. The RMSD graph (Figure S13a) for MAO‐A in complex with **Cy7‐MAO** shows that the system stabilizes before 50 ns and subsequently remains stable with very little fluctuation. These values indicate that the complex exhibits good stability. In the case of MAO‐B in complex with **Cy7‐MAO** (Figure S13b), similar behavior is observed, with rapid stabilization of the system and subsequent small fluctuations.

**FIGURE 4:**
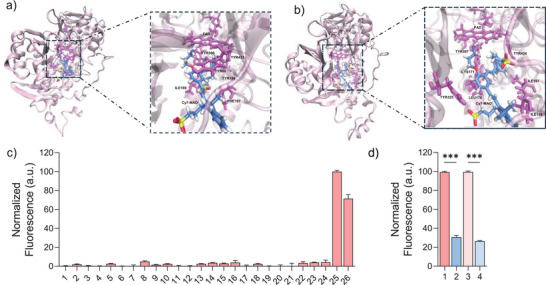
Molecular docking and selectivity studies. Interaction of **Cy7‐MAO** with isozymes MAO‐A and MAO‐B. (a) The MAO‐A enzyme is depicted in Cartoon form, and the active site residues are displayed in stick form. **Cy7‐MAO** (blue) is buried in the substrate binding pocket of the protein. The substrate binding residues are in the form of sticks (purple) and are labeled for a better illustration. (b) The MAO‐B enzyme is depicted in Cartoon form, and the active site residues are displayed in stick form. **Cy7‐MAO** (blue) is buried in the substrate binding pocket of the protein. The substrate binding residues are in the form of sticks (purple) and are labelled for a better illustration. (c) Fluorescence emission of **Cy7‐MAO** (5 µm) in HEPES (10 mm, pH=7.4, 37°C) in the presence of different potential interferents at 535 nm (excitation at 450 nm): 1, Na^+^ (1 mm); 2, K^+^ and NO_3_^−^ (1 mm); 3, Ca^2+^ (1 mm); 4, Mg^2+^ (1 mm); 5, Fe^2^^+^ (0.2 mm); 6, Fe^3^^+^ (0.2 mm); 7, H_2_O_2_ (10 mm); 8, t‐BuOOH (100 µm); 9, Vitamin C (1 mm); 10, Glutathione (1 mm); 11, Arginine (1 mm); 12, L‐Histidine (1 mm); 13, L‐Glutamic acid (1 mm); 14, Urea (20 mm); 15, Glucose (10 mm); 16, BSA (500 µm); 17, Lysozyme (150 mg·mL^−^^1^); 18, Acetyl Cholinesterase (150 mg·mL^−^^1^); 19, Leucine Aminopeptidase (150 mg·mL^−^^1^); 20, β‐Galactosidase (150 mg·mL^−^^1^); 21, Phosphatase (150 mg·mL^−^^1^); 22, Esterase (150 mg·mL^−^^1^); 23, DNase (150 mg·mL^−^^1^); 24, Glucose Oxidase (150 mg·mL^−^^1^); 25, MAO‐A (100 µg·mL^−^^1^) and 26, MAO‐B (100 µg·mL^−^^1^). (d) Fluorescence intensity: 1: **Cy7‐MAO** + MAO‐A; 2: **Cy7‐MAO** + MAO‐A + CL (clorgyline, a specific MAO‐A inhibitor); 3: **Cy7‐MAO** + MAO‐B; 4: **Cy7‐MAO** + MAO‐B + PA (pargyline, a specific MAO‐B inhibitor). The solutions used were prepared in HEPES (10 mm, pH=7.4, 37°C) with the following concentrations: **Cy7‐MAO** (10 µm), MAO‐A/B (10 µm), CL, and PA (10 µm). Error bars are expressed as 3*σ* for three independent experiments. Values are expressed as mean ± SD. Statistical analysis was assessed by applying Student's *t*‐test (^***^ *p* < 0.001).

The MD simulations indicate there is little movement at both binding sites, and a representative binding mode for **Cy7‐MAO** in complex with MAO‐A can be seen in Figure 4a, while the one with MAO‐B is shown in Figure 4b. In both cases, the amino group of **Cy7‐MAO** is facing the N5 and the carbonyl group of the FAD catalytic site. One reason for the stabilization of the probe in the active site cavity is most likely related to the presence of two Tyr residues that form an aromatic cage. In the case of MAO‐A, these are Tyr433 and Tyr396 (Figure S14a), and in the case of MAO‐B, Tyr434 and Tyr397 (Figure S14b). The results of the computer simulation agree with the experimental results and help explain how **Cy7‐MAO** binds to the MAO‐A and MAO‐B isoforms of the enzyme for the catalytic oxidation of the monoamine in **Cy7‐MAO** to give Cy7.

The selectivity of **Cy7‐MAO** probe to detect MAO‐A and MAO‐B was also assessed in the presence of several potentially interfering species [44]. As showed in Figure 4c, only both MAO isoforms induced a remarkable emission enhancement in **Cy7‐MAO**, whereas no changes were found in the presence of selected ions (Na^+^, K^+^, NO_3_^−^, Ca^2+^, Mg^2+^, Fe^2+^, Fe^3+^), small molecules (H_2_O_2_, vitamin C, arginine, urea, glucose, glutathione, L‐Histidina, L‐Glutamic acid) or other enzymes (acetyl cholinesterase, leucine aminopeptidase, β‐galactosidase, phosphatase, esterase, DNase, glucose oxidase). Besides, additional experiments were performed in the presence of MAO enzymes and the MAO inhibitors clorgyline for MAO‐A and pargyline for MAO‐B [24, 45]. In both cases, the fluorescence signal in the presence of inhibitors was 70% lower than the signal in its absence, confirming the proposed mechanism; i.e., the emission enhancement is due to the hydrolysis of **Cy7‐MAO** by MAO enzymes (Figure 4d).

### 2.3 In vitro Validation of the **Cy7‐MAO** Probe

**FIGURE 5:**
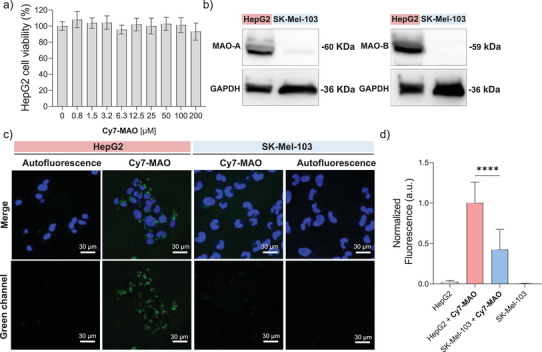
In vitro validation of **Cy7‐MAO** probe. (a) Cell viability in HepG2 cells incubated with different concentrations of **Cy7‐MAO** probe for 48 h. (b) Western blot assay for MAO‐A/B expression in HepG2 and SK‐Mel‐103 cells. The values are expressed as mean ± SEM (*n* = 3). (c) Confocal images of HepG2 (high endogenous expression of MAO) vs. SK‐Mel‐103 (control cells) incubated with **Cy7‐MAO** (100 µm) for 2 h. Nuclei were stained with Hoechst 33342 (blue channel). Scaler bar: 30 µm. (d) Fluorescence quantification of confocal images. Images show a 2‐fold emission enhancement in HepG2 cells when compared with SK‐Mel‐103 cells. The results exhibited representative images from three independent studies (*n* = 3), and values are expressed as mean ± SD. Statistical analysis was assessed by applying Student's *t*‐test (^****^ *p* < 0.0001).

For in vitro validation, human‐derived HepG2 (hepatocellular carcinoma) cells were selected for their known overexpression of both MAO isoforms [46]. Initially, **Cy7‐MAO** toxicity was tested in HepG2 cells, showing no toxic effect after 48 h of incubation, even at concentrations as high as 200 µm (Figure 5a). Western blot confirmed MAO expression in HepG2, showing endogenous high levels of MAO‐A and MAO‐B (Figure 5b). In contrast, SK‐Mel‐103 melanoma human‐derived cells, chosen as a negative control, showed minimal levels of MAO‐A and MAO‐B enzymes (Figure 5b). Confocal images of cells incubated with **Cy7‐MAO** probe showed a strong fluorescence signal in HepG2 cells compared to SK‐Mel‐103 cells (Figure 5c,d). These results confirm the selective hydrolysis of **Cy7‐MAO** in HepG2, supporting the MAO‐dependent activation of the probe in vitro.

### 2.4 In vivo Validation of **Cy7‐MAO** Probe

**FIGURE 6:**
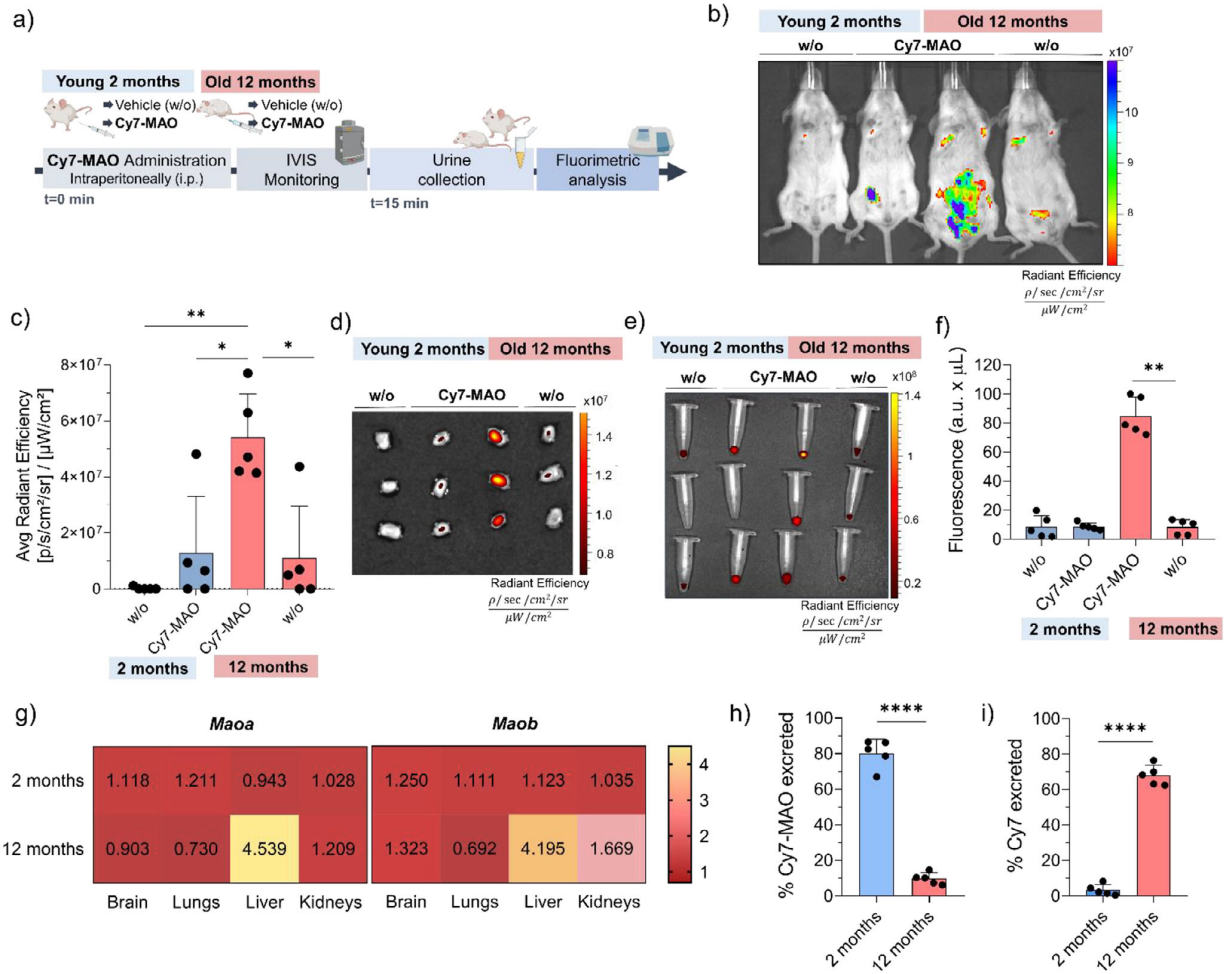
In vivo validation of **Cy7‐MAO** probe in a naturally aged mouse model. (a) Timeline for the in vivo evaluation of **Cy7‐MAO** probe in naturally aged BALB/cByJ mice model (2‐ and 12‐month mice, *n* = 5 animals per group). (b) Representative IVIS images of BALB/cByJ mice i.p injected with **Cy7‐MAO** and (c) quantification. (d) Ex vivo IVIS image of bladder from young (2 months) and old (12 months) after **Cy7‐MAO** or vehicle treatment (w/o). The image shows that only the bladder from old mice treated with **Cy7‐MAO** exhibits a strong fluorescence signal. (e) Representative IVIS image of urine samples from young (2‐month) and old (12‐month) mice recorded 15 min after **Cy7‐MAO** or vehicle treatment (w/o). (f) Fluorimeter measurement of Cy7 fluorophore in the urine of BALB/cByJ mice of different ages. Fluorescence values were normalized considering total urine volume (a.u. x mL) to avoid variability in micturition and ensure accurate comparison between groups. The values are expressed as mean ± SD. Statistical significance was assessed by the one‐way ANOVA followed by a Tukey's test: ^****^ *p* < 0.0001. (g) Heat map of MAO expression in main organs corroborated by real‐time qPCR. Values represent the mean of relative MAO mRNA expression (*n* = 5). *Actb* (β‐actin) was used for input normalization. (h) **Cy7‐MAO** (%) and (i) Cy7 (%) excreted through urine from BALB/c mice measured by HPLC‐MS. Values are expressed as mean ± SD. Statistical analysis was assessed by applying one‐way followed by a Tukey's test: ^****^ *p* < 0.0001.

The fact that MAO‐A and/or MAO‐B enzyme levels are frequently elevated with age has drawn attention to their potential contribution of MAO enzymes to physiological aging. Accumulating evidence indicates that MAO enzyme levels rise with age in various tissues, including the brain and liver, contributing to increased ROS production and mitochondrial dysfunction [47, 48]. These processes are considered key mechanisms in physiological aging and age‐related pathologies [49]. Given this established relationship, we were interested in evaluating the ability of **Cy7‐MAO** for non‐invasive readout of MAO in the context of aging. To this end, we conducted two complementary in vivo experiments using BALB/cByJ mice. In the first experiment, focused on mechanistic validation of the probe, we performed a comparative analysis between two distinct age groups, young (2 months) and aged (12 months), to explore functionality and specificity of the probe in detecting age‐dependent MAO differences. Mice were intraperitoneally administered with 10 mm **Cy7‐MAO**, and the fluorescence emission was monitored by IVIS (In Vivo Imaging System) technique (Figure 6a). In vivo IVIS imaging after 15 min showed a fluorescent signal around the peritoneal area and the anatomical region corresponding to the bladder in the older mice injected with **Cy7‐MAO** (Figure 6b). For quantification purposes, this specific region of interest (ROI), corresponding to the bladder zone, was consistently used across all animals to assess the relative fluorescence signal (Figure 6c), confirming the elevated fluorescence intensity in older mice. This pattern reflects the design of **Cy7‐MAO** as a renal‐clearable activatable probe: upon MAO‐dependent activation, the fluorescent Cy7 product is rapidly filtered by the kidneys and transiently concentrated in the bladder prior to urine elimination.

After urine collection and euthanasia, the fluorescence of the main organs was studied by IVIS imaging. The strong signal in the bladder of aged animals, compared to young mice (negligible signal), also reflects that **Cy7‐MAO** activation occurs in vivo in an age‐dependent manner, with fluorescence in the bladder reflecting efficient clearance of the processed probe via the renal pathway (Figure 6d). Moreover, fluorescence readout of urine, collected after mice recovered from anesthesia, correlates with this outcome. Urine samples were collected 15 min after intraperitoneal administration of the **Cy7‐MAO** probe and analysed at this reduced time interval to specifically evaluate the fluorescence of the Cy7 fluorophore. Aged mice showed a 5.8‐fold increase in urine fluorescence emission from fluorimeter measurements compared to young mice (Figure 6e,f), consistent with higher in vivo C**y7‐MAO** activation. Overall, these findings indicate that **Cy7‐MAO** is preferentially activated in aged animals, suggesting that the observed urinary fluorescence reflects age‐dependent increases in MAO. To corroborate that, we characterised *Maoa* and *Maob* mRNA levels in brain, lungs, liver, kidneys, and spleen by RT‐qPCR in 2 and 12‐month‐old mice, which serve as a proxy for enzyme expression. Previous studies using quantitative enzyme radioautography in BL6/C57 mice have reported organ‐specific, age –related changes in MAO [50]. In our experiments, a significant age‐dependent increase in both isoforms was observed in the liver, with *Maoa* and *Maob* levels rising approximately 4.5‐ and 4‐fold in 12‐month old mice, respectively (Figure 6g; Figure S16). In contrast, MAO expression remained unchanged in the other examined organs, except for kidneys, which also showed some slight increase in *Maoa*, and specially in *Maob*, levels in 12‐month‐old mice. In contrast, young mice showed no significant changes in MAO expression across these organs. These results corroborate that the elevated urinary fluorescence observed in aged mice reflects biologically relevant specific increases in MAO expression (both isoforms), while young mice do not exhibit such activation. Although MAO overexpression was widely associated with the brain, particularly in the context of neurodegenerative conditions, its expression in this tissue is known to be variable and context dependent [51]. On the other hand, our observations align with previous studies suggesting the liver as a major site of peripheral MAO activity in aging [25, 52, 53]. This enzymatic upregulation is consistent with reported age‐related hepatic alterations. This can be associated with the elevated oxidative stress and impaired metabolic capacity of the liver relative to other organs. This can lead to chronic inflammation, fibrosis, and increased expression of ROS–generating enzymes such as MAO.

Consistent with this, HPLC‐MS analysis performed on urine collected 15 min after probe administration confirmed activation of **Cy7‐MAO,** directly linking probe processing to the biological target and demonstrating efficient renal elimination of the activated product. In aged mice (12 months old), approximately 69.6 ± 5.7% of the administered dose of **Cy7‐MAO** (38 mg/kg) was recovered in the urine as the Cy7 fluorophore, while 4.1 ± 3.3 % remained as unprocessed **Cy7‐MAO** probe (Figure 6h,i). In contrast, in young mice (2 months old), excretion of the Cy7 fluorophore was minimal (ca. 1.5 ± 1.0%), whereas the **Cy7‐MAO** probe accounted for approximately 80.1 ± 8.1%. These percentages were calculated by relating the amounts of probe and fluorophore detected in urine to the total dose of **Cy7‐MAO** initially administered, reflecting rapid MAO‐mediated activation followed by renal clearance. These results confirm that **Cy7‐MAO** functions as designed, being selectively activated only in the presence of MAO in aged mice and rapidly cleared with high efficiency providing a robust, non‐invasive readout of age‐associated MAO expression. The remaining fraction likely represents transient circulation or distribution in non‐target tissues, without affecting probe specificity or reliability of the urinary readout.

**FIGURE 7:**
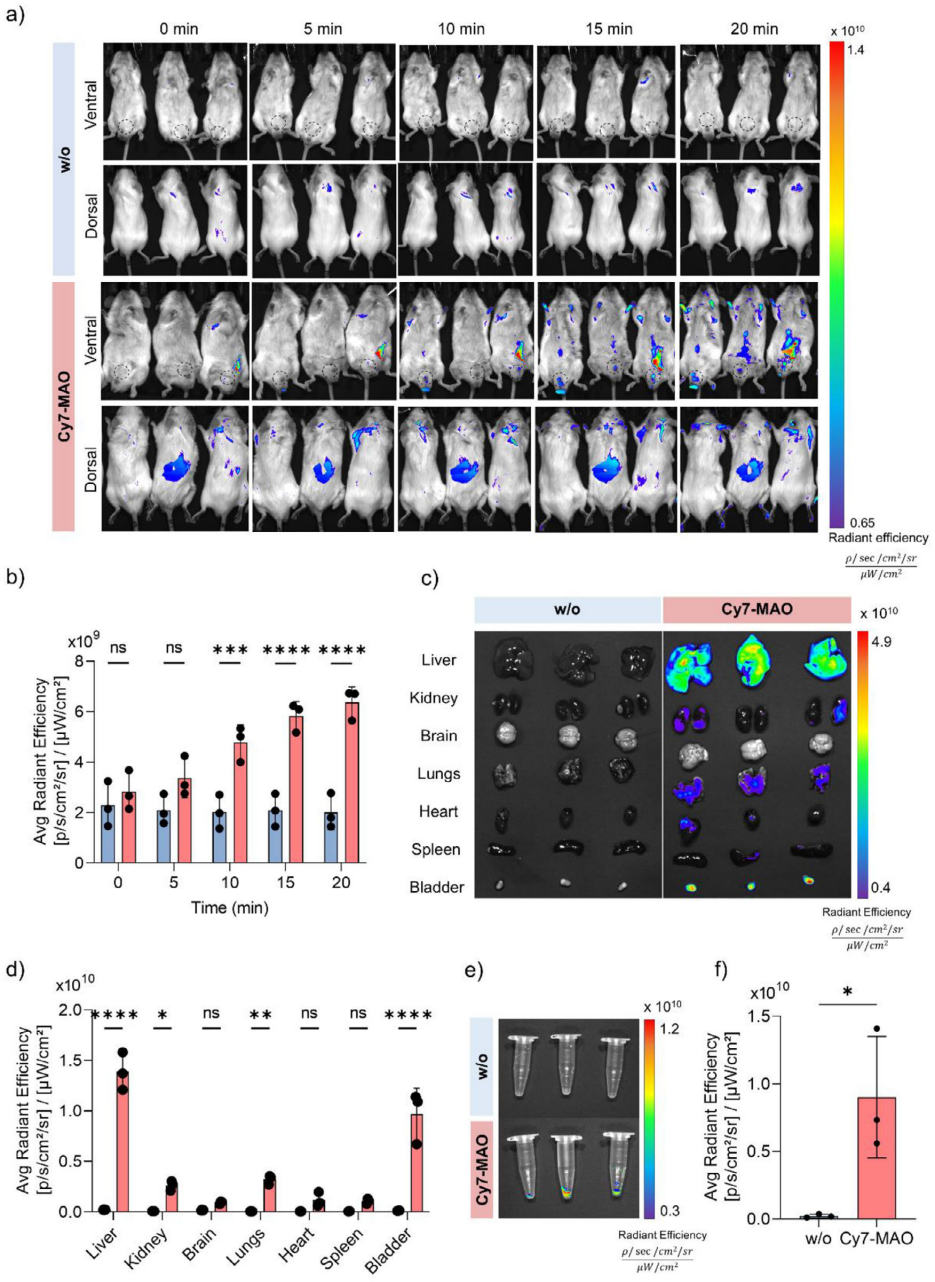
Functional distribution analysis of **Cy7‐MAO** in naturally aged mice. (a) AniView Phoenix obtained images of **Cy7‐MAO** (12‐month‐old) and vehicle‐treated mice after 0, 5, 10, 15, and 20 min in dorsal and ventral position. (b) Fluorescence (avg. Radiant effiency) quantification of bladder region in ventral images at different times. Images were taken before urine collection. (c) Ex vivo AniView Phoenix images and (d) quantification of dissected tissue of **Cy7‐MAO** (12‐month‐old) (pink bars) or vehicle (blue bars) treatment after 15 min and quantification. Ex vivo images were taken after urine collection. Data is represented as mean ± SD (*n* = 3). Statistical significance was assessed by applying two‐way ANOVA followed by a multiple‐comparison test. (e) AniView Phoenix images and (f) fluorescence quantification of urine collected from mice injected with **Cy7‐MAO** (pink bars) or vehicle (blue bars). Data is represented as mean ± SD (*n* = 3). Statistical significance was assessed by applying unpaired *t*‐test: ^*^ *p* < 0.05.

In a complementary study, a more detailed functional distribution analysis in aged mice was performed. In vivo imaging monitoring showed the dynamic transit of the probe from the injection site to the urinary system over 0–20 min (Figure 7a). Quantification of the in vivo fluorescence signal showed a clear increase in the bladder region, reflecting the high local concentration of the activated Cy7 fluorophore (Figure 7b). In vivo imaging was performed before urine collection, which is consistent with a high concentration of the activated Cy7 in the bladder. Ex vivo imaging (Figure 7c), performed after urine collection (Figure 7e,f), revealed a portion of residual fluorescence predominantly in the liver and in the bladder with negligible signal in other organs. These patterns do not reflect accumulation or a conventional biodistribution but rather confirm that the activated probe is efficiently cleared via urine, with residual signals arising from sites of enzymatic activation (e.g., liver) or incomplete urinary elimination. The results are consistent with the design of **Cy7‐MAO** as a renal‐clearable activatable probe: upon MAO‐dependent activation, the fluorescent Cy7 product is rapidly processed by the renal system, and temporarily concentrated in the bladder prior to urine elimination. Importantly, acute toxicity studies in healthy mice indicated no detectable adverse effects on well‐being, blood chemistry, or organ histology, supporting the biocompatibility of the probe at the tested dose (Figure S17).

To further investigate whether **Cy7‐*MAO*** could detect more gradual, age‐associated changes in MAO activity and support longitudinal studies, a second experiment was carried out with mice at intermediate ages. Specifically, BALB/cByJ mice aged 2 (*n* = 3), 5 (*n* = 3), 8 (*n* = 3), and 14 (*n* = 2) months were intraperitoneally administered with **Cy7‐MAO** (10 mm), and the urine was collected 15 min after the mice recovered from anesthesia for fluorescence analysis (Figure 7a).

**FIGURE 8:**
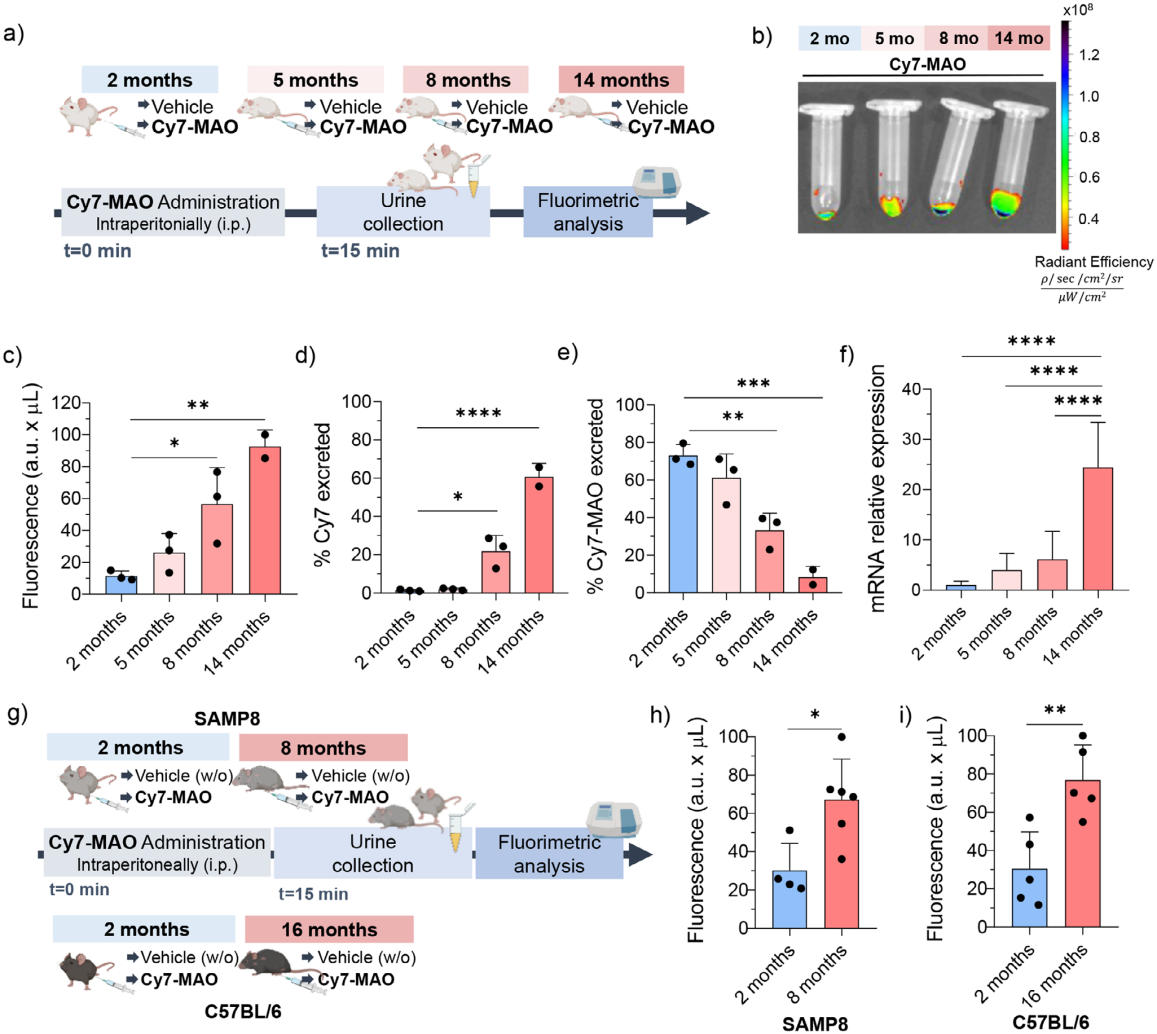
In vivo validation of **Cy7‐MAO** probe across aging progression and mouse models. (a) Timeline for the in vivo evaluation of **Cy7‐MAO** probe in naturally aged BALB/cByJ mice. (b) Representative IVIS image of urine samples from 2‐, 5‐, 8‐, and 14‐months mice recorded 15 min after **Cy7‐MAO**. (c) Fluorimeter measurement of Cy7 fluorophore in the urine of BALB/cByJ mice of different ages. Fluorescence values were normalized considering total urine volume (a.u. x mL) to avoid variability in micturition and ensure accurate comparison between groups. The values are expressed as mean ± SD. Statistical significance was assessed by the one‐way ANOVA followed by a Tukey's test: ^**^ *p* < 0.01. (d) Measurement of the percentage of Cy7 fluorophore excreted in the urine by HPLC‐MS. The values are expressed as mean ± SD. Statistical significance was assessed by the one‐way ANOVA followed by a Tukey's test: ^****^ *p* < 0.0001. (e) Measurement of the percentage of **Cy7‐MAO** probe excreted in the urine by HPLC‐MS. The values are expressed as mean ± SD. Statistical significance was assessed by the one‐way ANOVA followed by a Tukey's test: ^***^ *p* < 0.001. (f) mRNA expression levels of *Maoa* of *Cdkn2a* (which encodes for p16^Ink4a^) in liver corroborated by real‐time PCR. *Actb* was used for input normalization. Values are relative to control mice and are expressed as mean ± SD. Statistical significance was assessed by the one‐way ANOVA followed by a Tukey's test: ^*^ *p* < 0.05; ^**^ *p* < 0.01; ^***^ *p* < 0.001; ^****^ *p* < 0.0001 (*n* = 3). (g) Timeline for the in vivo evaluation of **Cy7‐MAO** probe in SAMP8 and C57BL/6 mice. (h) Fluorimeter measurement of Cy7 fluorophore in the urine of SAMP8 and (i) C57BL/6 mice of different ages. Fluorescence values were normalized considering total urine volume (a.u. x mL) to avoid variability in micturition and ensure accurate comparison between groups. The values are expressed as mean ± SD. Statistical significance was assessed by the one‐way ANOVA followed by a Tukey's test, ^*^ *p* < 0.05; ^**^ *p* < 0.01.

Urine fluorescence measurements revealed an increasing trend in fluorophore emission with mice age (Figure 8b,c). As described above, given the short post‐injection interval (15 min), the fluorescence signal corresponds to the formation of the Cy7 fluorophore and not to the hemiCy7 derivative. A significant difference was observed in the emission intensity between the youngest (2‐month‐old) and the oldest (14‐month‐old) mice (ca. 6.5‐fold), with intermediate values for mice aged 5 and 8 months. These results suggest that **Cy7‐MAO** is sufficiently sensitive to detect age‐associated differences in MAO levels through urinary fluorescence. HPLC‐MS analysis in urine revealed values of Cy7 fluorophore that increased with age from 1.5 ± 0.5% for 2‐month‐old mice to 62.7 ± 7.1 % for 14‐month‐old mice (Figure 8d). HPLC‐MS analysis also demonstrated that the remaining **Cy7‐MAO** probe that is not hydrolysed is also excreted through the urine with values ranging from 76.4 ± 5.9% to 8.2 ± 5.7 % of the injected probe for 2‐ and 14‐month‐old mice (Figure 8e). Intermediate age groups (5 and 8 months) showed progressive increases of Cy7, and a decrease of **Cy7‐MAO** compared to 2‐month‐old mice. These observations are consistent with the fact that younger animals, which exhibit lower MAO enzymatic activity, excrete a larger proportion of the **Cy7‐MAO** probe. In contrast, older animals, with higher MAO activity, show extensive conversion of **Cy7‐MAO** into the fluorescent Cy7 product. This age‐dependent differential activation provides a clear dynamic window, demonstrating that the probe can effectively discriminate variations in MAO activity across different ages. As in the previous study, these data indicate that a large amount of the injected probe is eliminated in the urine (as Cy7 or **Cy7‐MAO**) after only 15 min, suggesting minimal retention in tissues or elimination via alternative pathways, and confirming renal excretion as the main clearance route. Additionally, to further confirm the physiological aging context in the liver as suggested in the study above, hepatic expression levels of the senescence marker p16^Ink4a^ (encoded by the *Cdkn2a* gene) were evaluated across the different age groups. A progressive increase in *Cdkn2a* levels was observed with age, with a marked expression in 14‐month‐old mice (Figure 8f), thereby linking the observed **Cy7‐MAO** activation to an aging phenotype. Overall, these results corroborate the potential of the **Cy7‐MAO** probe as a minimally invasive tool for detecting biologically relevant MAO‐dependent differences in aging, offering complementary insights beyond static gene expression assays.

Inspired by the above results, the **Cy7‐MAO** probe was also validated in additional mouse models of different ages, such as the senescence‐prone SAMP8 (2 and 8 months) [54] and the naturally aging C57BL/6 strain (2 and 16 months) (Figure 8g), following a similar procedure to that described above. Urine analysis revealed a significant increase in fluorescence emission with age in both strains. Specifically, an approximately 2.2‐fold increase in fluorescence intensity was observed between 2‐ and 8‐month‐old SAMP8 mice (Figure 8h), and a 2.8‐fold increase was detected between 2‐ and 16‐month‐old C57BL/6 mice (Figure 8i). These findings indicate that the age‐related increase in MAO is consistent across different mouse strains and observed in both natural (BALB/cByJ and C57BL/6) and accelerated aging (SAMP8) models, highlighting the versatility of **Cy7‐MAO** as a tool for detecting MAO‐dependent changes in vivo with age.

Overall, these results demonstrate the potential use of the **Cy7‐MAO** as a reliable and non‐invasive fluorescent probe for monitoring MAO activity in vivo, as evidenced by the clear correlation between age‐related increases in MAO activity and urinary fluorescence.

## 3 Conclusions

In the context of precision medicine, one attractive approach is to detect biomarkers from accessible biofluids using simple detection systems to guide healthcare decisions. One approach toward this aim is the design of probes in an OFF state that can be transformed by specific biomarkers in cells or tissues in vivo to give an ON, highly emissive product that can be renally cleared, allowing fluorescence detection in urine by simple techniques. Based on this concept, we report herein a fluorogenic molecular probe (**Cy7‐MAO**) to selectively detect MAO enzymes through fluorescence measurements in collected urine samples. MAO enzymes induce the oxidative deamination of the poorly fluorescent **Cy7‐MAO** probe, generating the highly emissive Cy7 fluorophore, which evolves with time to the also emissive hemiCy7. Confocal studies confirmed the ability of **Cy7‐MAO** to detect MAO in vitro in HepG2, in which there is an endogenous MAO overexpression. We also provided evidence that urinary fluorescence correlates with relative levels of MAO in elderly and young BALB/c mice in vivo. We found that urine emission in 12‐month‐old mice was 5.8‐fold higher than in 2‐month‐old animals. These are directly related to the relative mRNA expression of *Maoa* and *Maob*, clearly higher in the liver of old mice. Importantly, biodistribution analysis confirmed that after injection, the probe transits through the liver and kidneys to the bladder, with urinary excretion as the main clearance route, and minimal fluorescence was detected in other major organs. This supports the conclusion that urinary fluorescence predominantly reflects liver‐associated MAO activity. Additionally, **Cy7‐MAO** also captured gradual age‐associated changes in MAO levels through urinary fluorescence in BALB/c mice of different ages. A progressive increase in *Cdkn2a* levels was also observed with age, thereby linking the observed **Cy7‐MAO** activation to an aging phenotype. Furthermore, validation in additional mouse strains (SAMP8 and C57BL/6) confirmed that this age‐associated increase in fluorescence is consistent across genetic backgrounds, supporting the broader applicability of the probe. Overall, the results highlight the value of **Cy7‐MAO** as a non‐invasive tool to monitor biologically relevant, MAO‐dependent changes in aging in vivo, previously not feasible with existing methods. This probe provides a simple and effective readout of MAO‐associated alterations, offering a new approach for studying conditions associated with elevated MAO levels and opening the way to linking MAO to its involvement in possible pathological contexts with age. This approach not only provides a simple and effective readout of MAO dysregulation but also underscores the broader applicability of renal‐clearable probes for the real‐time assessment of multiple metabolic and pathological conditions.

## Conflicts of Interest

The authors declare no conflicts of interest.

## Supporting information




**Supporting File**: advs74414‐sup‐0001‐SuppMat.docx

## Data Availability

The data that support the findings of this study are available from the corresponding author upon reasonable request.
