# Supplementary material for: A Renal Clearable Probe for In Vivo Monoamine Oxidase (MAO) Detection
Source: Adv Sci (Weinh). 2026 Feb 23;13(23):e20797. doi: 10.1002/advs.202520797 (PMC13104153; doi:10.1002/advs.202520797)
Supplement: Supplementary file 1 — Supporting File: advs74414‐sup‐0001‐SuppMat.docx [file ADVS-13-e20797-s001.docx]

A renal clearable probe for *in vivo* Monoamine Oxidase (MAO) detection

Marcia Domínguez,^a,b^ David Azorín-Soriano,^a,c^ Araceli Lérida-Viso,^a,b,c,d^ Jessie García-Fleitas,^a,b^ Paula M Soriano-Teruel^a,b,c,h^ Jennifer Soler- Beatty,^a,c^ Paula Rodrigo-Martínez,^d^ Pau Arroyo,^d,e^ Sara Rojas-Vázquez,^a,b,f^ Isabel Fariñas,^f,g^ Félix Sancenón,^a,b,c,h^ Juan F. Blandez,^i^ Alba García-Fernández,^a,b,c,h*^ and Ramón Martínez-Máñez,^a,b,c,h,*^

1. M. Domínguez, D. Azorín-Soriano, A. Lérida-Viso, J. García-Fleitas, P.M. Soriano-Teruel, J. Soler- Beatty, S. Rojas-Vázquez, F. Sancenón, J. F. Blandez, A. García-Fernández, R. Martínez-Máñez
   Instituto Interuniversitario de Investigación de Reconocimiento Molecular y Desarrollo Tecnológico (IDM), Universitat Politècnica de València, Universitat de València, Camino de Vera, s/n 46022, Valencia, Spain.
   E-mail: [rmaez@qim.upv.es](mailto:rmaez@qim.upv.es), [algarfe4@etsiamn.upv.es](mailto:algarfe4@etsiamn.upv.es)
2. M. Domínguez, A. Lérida-Viso, J. García-Fleitas, P.M. Soriano-Teruel, S. Rojas-Vázquez, F. Sancenón, A. García-Fernández, R. Martínez-Máñez
   CIBER de Bioingeniería, Biomateriales y Nanomedicina (CIBER-BBN), Instituto de Salud Carlos III.
3. D. Azorín-Soriano, A. Lérida-Viso, P.M. Soriano-Teruel, F. Sancenón, A. García-Fernández, R. Martínez-Máñez
   Unidad Mixta UPV-CIPF de Investigación en Mecanismos de Enfermedades y Nanomedicina, Universitat Politècnica de València, Centro de Investigación Príncipe Felipe, C/Eduardo Primo Yúfera 3, 46100 Valencia, Spain.
4. P. Rodrigo-Martínez, P. Arroyo Instituto Interuniversitario de Investigación de Reconocimiento Molecular y Desarrollo Tecnológico, Universitat de València, Universitat Politècnica de València, C/ Doctor Moliner, 50, Burjassot, 46100, Valencia, Spain.
5. P. Arroyo Departamento de Química Orgánica, Universitat de València, C/Doctor Moliner, 50, Burjassot, 46100, Valencia, Spain.
6. S. Rojas-Vázquez, I. Fariñas Instituto de Biotecnología y Biomedicina (BIOTECMED), Universitat de València, Valencia, Spain.
7. I. Fariñas CIBER de Enfermedades Neurodegenerativas (CIBERNED), Instituto de Salud Carlos III, 28029 Madrid.
8. A. Lérida-Viso, P.M. Soriano-Teruel, J. Soler- Beatty, F. Sancenón, J. F. Blandez, A. García-Fernández, R. Martínez-Máñez
   Unidad Mixta de Investigación en Nanomedicina y Sensores, Universitat Politècnica de València, Instituto de Investigación Sanitaria La Fe (IIS La Fe), Avenida Fernando Abril Martorell, Torre A, Laboratorio. 7.32, 46026, Valencia, Spain.
9. J. F. Blandez
   Instituto de Ciencia Molecular (ICMol), Universidad de Valencia, C/Catedrático José Beltrán Martínez, 2, 46980 Paterna, Valencia, Spain.

**Table S1.** Comparison of **Cy7-MAO** with other fluorescent probes for the detection of MAO overexpression.

| **Probe** | **λ _exc_/ λ _em_ (nm)** | **LOD** | **Selectivity** | ***In vitro* (Cell lines)** | ***Animal model*** | **Detection time (*In Vitro*/ *In Vivo*)** | **Ref.** |
| --- | --- | --- | --- | --- | --- | --- | --- |
| **MR1/MR2** | 544/590 | Not reported | MAO-A/B | PC12 | Not reported | 7 h/ Not reported | Albers *et al*. *Chem. Comm.* **2007**, *44*, 4647-4649 |
| **IminoPOS** | 448/585 | Not reported | MAO-A/B | C6 glioma | Not reported | 1 h/ Not reported | Kim *et al.* *Chem. Comm.*  **2012**, *48*, 6833-6835. |
| **U1** | 304/449 | Not reported | MAO-B | HepG2  SH-SY5Y | Drosophila  Brain of mice  (*ex vivo*) | 2 h/ 3 h | Li *et al.* *Nat.Comm*. **2014**, 3276 |
| **Probe 1-4** | 470/535 | 3.5 µg /mL (MAO-A)  6.0 µg /mL (MAO-B) | MAO-A/B | MCF-7 | Not reported | 3 h/ Not reported | Li *et al.* Org. *Biomol. Chem.* **2014**, 12, 2033-2036. |
| **Probe 1-2** | 475/570 | 34.7 µg/ mL | MAO-A/B | HepG2, HeLa,  MCF-7, RAW,  SH-SY5Y, BV2 | Not reported | 2 h/ Not reported | Li *et al.* *Analyst*.  **2014**, 139, 6092-6095 |
| **M2** | 410/510 | Not reported | MAO-B | HepG2  SHSY5Y | Not reported | 1 h/ Not reported | Li *et al.* *Angew. Chem. Int. Ed.* **2015**, 54, 10821-10825 |
| **MAO-Red-1/2** | 420 /664 | 1.2   μg/ mL | MAO-A/B | HeLa HepG2 | Not reported | 1 h / Not reported | Li *et al*, *Sci. Rep.* **2016**, 6:31217, 1-8 |
| **Probe 1** | 425/550 | 1.1 ng/mL | MAO-A | HeLa  NIH-3T3 | Not reported | 1 h/ Not reported | Wu *et al*. *Anal. Chem.* **2016**, 88, 1440-1446 |
| **Probe 3** | 550/586 | 2.7 ng/mL | MAO-A | SY-SY5Y  HepG2 | Not reported | 3 h/ Not reported | Wu *et al*. *Angew. Chem., Int. Ed.* **2017**, 56, 15319-15323 |
| **MitoHCy-NH_2_** | 730/770 | Not reported | MAO-B | HepG2  SMMC7721 | BALB/c mice  (*ex vivo* and *in vivo*) | 100 min/ 30 min | Wang *et al*. *Anal. Chem.* **2018**, 90, 4054-4061 |
| **Probe 3** | 530/675 | 2.6 ng/mL | MAO-A | SH-SY5Y NIH-3T3 HepG2 | Tumor-bearing nude mice  (*in vivo*) | 120 min/ 120 min | Yang *et al*. *Chem. Commun.* **2019**, 55, 2477 |
| **F1/FD1** | 430/618 | Not reported | MAO-A | SY-SY5Y | Mice  Tissues of human  (*ex vivo*) | 2 h/ Not reported | Fang *et al*, *Angew. Chem. Int. Ed.* **2020**, 59, 7536-7541 |
| **Probe A** | 425/550 | 1.1 ng/mL | MAO-A | Hela | Zebrafish  (*in vivo*) | 60 min / 30 min | Meng *et al*. *Dyes Pigments*. **2020**,176, 108208 |
| **DHMP2** | 680/710 | 13.0 ng/mL | MAO-A | SH-SY5Y  PC-3 | Zebrafish  Tissues of rats/mice  (*in vivo*) | 12 h/ 12 h | Yang *et al*, *ACS Sens*. **2020**, 5, 943–951 |
| **Rma-1/2** | 680/708 | 4.5 ng/mL (MAO-A)  13-fold (MAO-B) | MAO-A/B | HeLa | Zebrafish  Tumor-bearing BALB/c mice  (*in vivo*) | 1 h/ 30 min | Shang *et al*. *Anal. Chem.* **2021**, 93, 4285−4290 |
| **HCCP** | 540/626 | Not reported | MAO-A | U251  Hela | Tissues of mice  (*ex vivo*) | 24 h/ Not reported | Wu *et al*. *Anal. Chim. Acta.* **2022**, 1199,339573 |
| **AIE-M** | 430/600 | 46 ng/mL | MAO-A | SH-SY5Y NIH-3T3 | Zebrafish Imaging | 2 h/ 2h | Yang *et al.* Mater. Today Chem. **2024**, 35, 101890. |
| **Cy7-MAO** | 450/550 | 6.9 µg/mL (MAO-A)  10.9 µg/mL  (MAO-B) | MAO-A/B | HepG2 | Naturally aged BALB/cByJ mice  (*in vivo*) | 2 h / 15 min through urine | This work |

1. *Materials and methods*

3-(Boc-amino) propyl bromide, potassium carbonate, 1-(4-sulfobutyl)-2,3,3-trimethylindolium inert salt (**2**), sodium acetate, sodium hydroxide, acetic anhydride, tertbutyldimethylsilyl chloride (TBSCl), dichloromethane, *N*,*N*-dimethylformamide (DMF), trifluoroacetic acid (TFA) and monoamine oxidase A and B (MAO-A and MAO-B) were obtained from Sigma-Aldrich and used without further purification. 5-formylsalicylaldehyde was purchased from Fluorochem. Anhydrous acetonitrile was obtained from Acros Organics. The synthesis of the Cy7 fluorophore was carried out according to a previously reported procedure [1].

^1^H and ^13^C NMR spectra were recorded on a Bruker FT-NMR Avance 400 (Ettlingen, Germany) spectrometer at 300 K. HRMS data were obtained with a TRIPLETOF T5600 (ABSciex, USA) spectrometer. HPLC-MS was recorded with an Agilent 1620 Infinity II HPLC coupled to a mass spectrometer Agilent Ultivo equipped with a triple QTOF detector. Fluorescence spectroscopy was carried out in a JASCO spectrofluorometer FP-8500 and absorption spectra were collected in a JASCO V-650 spectrophotometer. Confocal fluorescence images were taken on a Leica TCS SP8 AOBS. Images were analyzed using ImageJ software. Monitoring of fluorescence in animals was carried out in an IVIS Spectrum *In Vivo* Imaging System (PerkinElemer), and images were analyzed by using the Living Image software.

1. *Synthesis and characterization of* ***Cy7-MAO*** *molecular probe*
   1. *Synthesis and characterization of 4-(3-aminopropoxy) isophthalaldehyde (****1****).*

5-formylsalicylaldehyde (500 mg, 3.3 mmol), sodium hydroxide (133 mg, 3.3 mmol), and 3-(Boc-amino) propyl bromide (786 mg, 3.3 mmol) were dissolved in anhydrous acetonitrile (5 mL). The reaction mixture was stirred under argon atmosphere at 70°C overnight. After that, TFA (5 mL DMF: 750 μL TFA) was added to the reaction mixture and stirred for 24 h. Solvent was removed under vacuum yielding product **1** as a yellow solid (457.5 mg, 91.5% yield). This product was used without further purification. ^1^H NMR (400 MHz, DMSO-d_6_) δ (ppm): 10^1^H NMR (400 MHz, DMSO-d_6_) δ (ppm): 10.71 (s, 1H), 10.20 (s, 1H), 8.53 (d, J = 2.3 Hz, 1H), 8.31 (dd, J = 8.8, 2.3 Hz, 1H), 7.43 (d, J = 8.7 Hz, 1H), 4.60–4.53 (m, 2H), 3.58 (td, J = 6.1, 2.3 Hz, 2H), 2.28–2.14 (m, 2H). ^13^C NMR (101 MHz, DMSO-d_6_) δ (ppm): 191.14, 190.21,165.24,135.85,131.97, 128.41, 122.69, 118.38, 66.37, 37.75, 35.92.

**Figure S1.** ^1^H-NMR of compound **1**.

Figure S2. ^13^C-NMR of compound 1.

- 1. *Synthesis and characterization of* ***Cy7-MAO****.*

**1** (250 mg, 1.2 mmol), commercially available 1-(4-sulfobutyl)-2,3,3-trimethylindolium) inner salt (**2**) (709 mg, 2.4 mmol) and sodium acetate (196.8 mg, 2.4 mmol) were dissolved in acetic anhydride (5 mL). The reaction mixture was heated to 70°C and stirred under argon atmosphere overnight. The solvent was removed by evaporation under reduced pressure, and the crude product was purified by silica gel chromatography eluted with hexane-ethyl acetate (v/v, 1:1), affording probe **Cy7-MAO** as yellow-brown oil (300 mg, 60% yield). ^1^H NMR (400 MHz, MeOD) δ ^1^H NMR (400 MHz, MeOD) δ 8.42 (dd, 1H), 8.21 (s, 1H), 8.02 (dd, J = 8.6, 2.2 Hz, 1H), 7.92 – 7.85 (m, 1H), 7.81 – 7.75 (m, 2H), 7.72 – 7.59 (m, 3H), 7.15 (t, J = 7.7 Hz, 2H), 7.01 (d, J = 7.3 Hz, 1H), 6.95 (d, J = 8.6 Hz, 1H), 6.76 (t, J = 7.4 Hz, 1H), 6.63 (d, J = 7.9 Hz, 1H), 4.72 (t, J = 8.1 Hz, 2H), 3.02 (t, J = 6.8 Hz, 2H), 2.80 (t, J = 7.5 Hz, 3H), 2.63 (s, 2H), 2.25 (d, J = 8.7 Hz, 4H), 2.11 (m, 4H), 1.88 (s, 12H).^13^C NMR (101 MHz, MeOD) δ (ppm): δ 13C NMR (101 MHz, MeOD) δ 196.98, 181.97, 160.97, 146.98, 143.62, 140.87, 139.31, 136.87, 135.26, 131.47, 129.20, 128.75, 127.49, 127.36, 122.62, 120.98, 119.66, 118.02, 116.20, 114.49, 109.81, 106.03, 54.63, 52.25, 49.59, 44.53, 26.88, 26.67, 26.42, 25.40, 25.36, 22.68, 22.21, 21.86.HRMS: Theoretical (C_39_H_47_N_3_O^2●2+^): 573.3708 m/z and (C_26_H_33_N_2_O_4_S^2●+^): 469.2156 m/z. Experimental (C_39_H_47_N_3_O_2_^●2+^): 572.1935 m/z and (C_26_H_33_N_2_O_4_S^2●+^): 470.1615 m/z respectively.

**Figure S3.** ^1^H-NMR of **Cy7-MAO**.


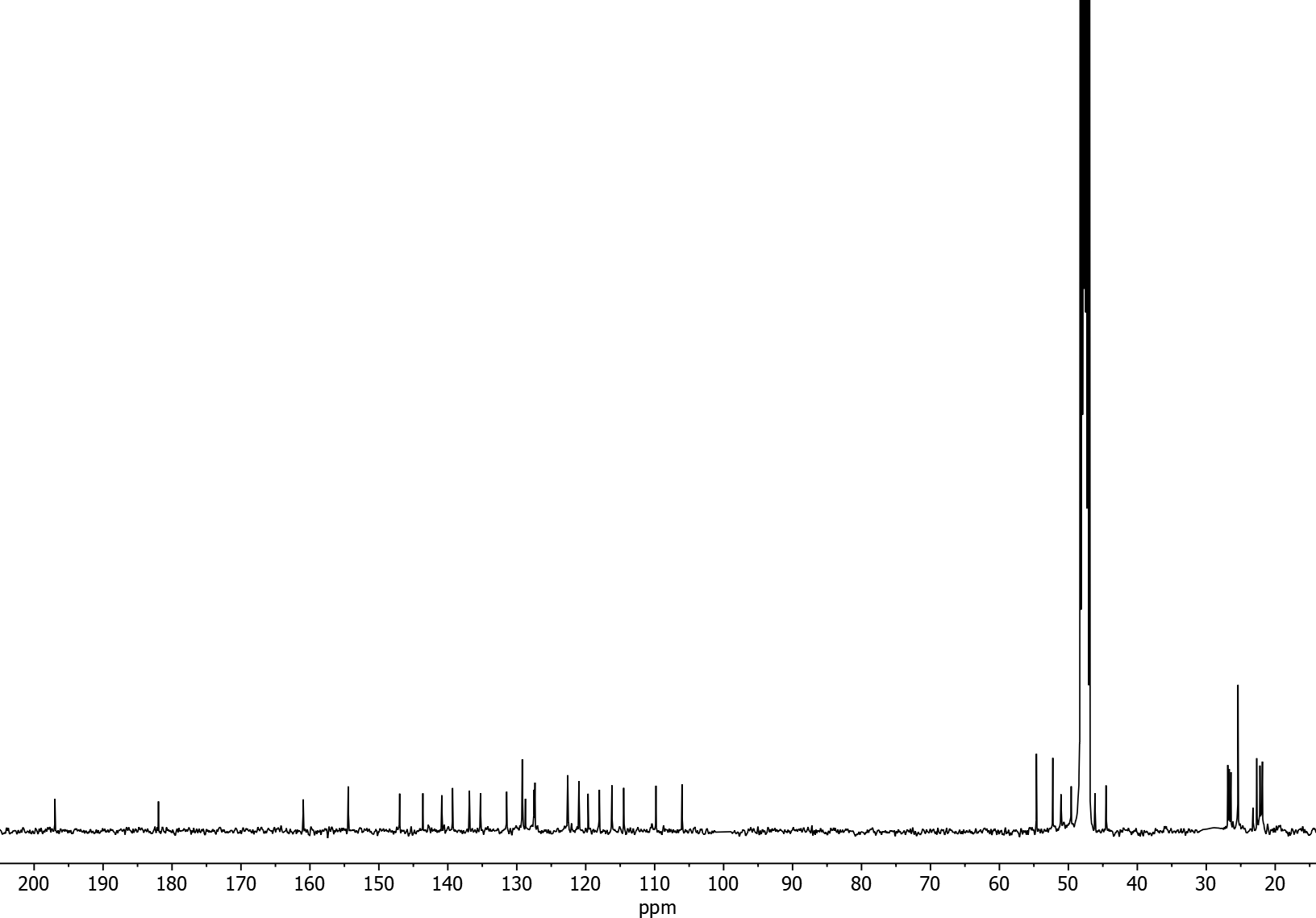


**Figure S4.** ^13^C-NMR of **Cy7-MAO**.

**Figure S5.** HRMS of **Cy7-MAO**.

1. *Synthesis of Cy7 fluorophore*

**Figure S6.** Synthetic route of Cy7 fluorophore.

1. *Cy7 fluorescence emission at different pH*

**Figure S7.** Emission intensity at 660 nm (λ_ex_ = 580 nm) of Cy7 fluorophore (5 µM) HEPES solutions at pH 5, 6, 7, 8, 9 and 10. Error bars are expressed as 3σ for three independent experiments.

1. *Characterisation of the* ***Cy7-MAO*** *probe and Cy7 fluorophore by HPLC-MS*

Figure S8. a) Mass spectrum of the Cy7-MAO probe obtained from the peak recorded at 11.01 minutes, at different time. A single 10^-3^ M dilution of Cy7-MAO was prepared, and aliquots were analysed by mass spectrometry at various time intervals to assess the stability of the Cy7-MAO probe. b) Mass spectrum of (a) showing a signal at 745 m/z corresponding to [M-CH_3_]^+^ of Cy7-MAO.

Figure S9. (a) Mass spectrum of the Cy7 fluorophore obtained from the peak recorded at 9.79 minutes, at different time intervals. A single 10^-3^M dilution of Cy7 was prepared and aliquots were analysed by mass spectrometry at various time intervals to assess the stability of the Cy7 fluorophore. b) Mass spectrum of (a) showing a signal at 705 m/z corresponding to [M+H^+^] of Cy7 fluorophore.

1. *Synthesis and characterization of hemiCy7.*

The synthesis and characterisation of the hemicy7 compound were carried out following the protocol previously described in the literature [2]. All experimental steps, as well as the purification and analytical conditions, were performed in accordance with the reported methodology without significant modifications.

**Figure S10.** Synthetic route of hemiCy7 fluorophore.

Figure S11. (a) UV-visible spectra of hemiCy7 10^-4^ M in HEPES (10 mM, pH 7.4). (b) Fluorescence spectra of hemiCy7 10^-4^ M in HEPES (10 mM, pH 7.4) upon excitation at 450 nm.

1. *General Procedure for MAO Detection*

Fluorescence emission measurements of **Cy7-MAO** were carried out with 1.0 μL of the probe from a stock solution (10^-3^ M in HEPES), followed by addition of MAO-A and MAO-B solution in HEPES (10 mM, pH 7.4). Final volume was adjusted to 200 µL with HEPES at pH 7.4. After incubation at 37°C for 24 h in a thermostat, solution was transferred to a quartz cell of 1 cm optical length to measure its fluorescence (λ_exc_ = 450 nm). A blank solution without MAO-A and MAO-B were prepared and measured under the same conditions.

1. *Calibration curve in HEPES solution.*

LOD and LOQ were obtained from the plot of fluorescence intensities at 535 nm (upon excitation at 450 nm) versus MAO-A/B concentration. LOD and LOQ were calculated by using the equation S1[3], where K=3 for LOD and K=10 for LOQ; Sb is the standard deviation of the blank and m is the slope of the calibration curve. The resulting LOD were 6.4 and 10.9 µg·mL^-1^ for MAO-A and MAO-B respectively. On the other hand, LOQ were 29.3 µg·mL^-1^ for MAO-A and 52.1 µg·mL^-1^ for MAO-B.

LOD/LOQ=K*Sb/m (Eq. S1)

1. *Study of Enzymatic Kinetics.*

For the determination of Michaelis-Menten constant (K_m_), the time-dependent fluorescence intensities of the reaction mixture of different concentrations of **Cy7-MAO** and MAO-A/B (100 µg·mL^-1^) were measured. The initial reaction velocities were then calculated. The relationship between the rate of enzymatic reaction and the concentration of **Cy7-MAO** can be expressed by the Michaelis-Menten equation:

$V_{0}=\frac{V_{max}[S]}{K_{m}+ [S]}$ (Eq. S2)

where Vo, Vmax, [S], and Km are the initial velocity, maximum velocity, substrate concentration, and Michaelis-Menten constant, respectively. K_m_ can be measured by using the Lineweaver-Burk plot with the following equation:

$\frac{1}{V_{0}}= \frac{K_{m}}{V_{max}[S]}+ \frac{1}{V_{max}}$ (Eq. S3)

where the slope is $\frac{K_{m}}{V_{max}}$ and y-intercept is $\frac{1}{V_{max}}$.

**Figure S12**. Lineweaver-Burk plot for the kinetic study of the reaction of different concentrations of **Cy7-MAO** whit MAO-A (a) and MAO-B (b) (100 µg·mL^-1^) in HEPES (10 mM, pH 7.4).

1. *Computational details*

The geometry of **Cy7-MAO** was optimized using density functional theory (DFT) at the B3LYP/6-31G(d,p) level as implemented in the Gaussian 16 software package [4]. All calculations were carried out in the gas phase without symmetry constraints. The three-dimensional X-ray crystal structures of MAO-A and MAO-B were retrieved from the Protein Data Bank (PDB ID 2Z5X [5] and 6YT2 [6], respectively). The characteristics of the substrate binding site were selected based on the ligand present in the X-ray structure to create the docking receptor grid. Docked structures were then analysed with AutoDock Tools [7] to determine the most favourable conformation based on binding energy and the population of the clusters obtained.

The molecular dynamics (MD) simulations were performed using the AMBER 22 software package [8] to investigate the binding strength, stability, and flexibility of the compound after docking. The ff19SB [9] force field was used for the protein, while the GAFF [10] force field was applied to the **Cy7-MAO** and the FAD cofactor. Parameters for FAD cofactor [11] were obtained from the AMBER parameter database from the Bryce Group (The University of Manchester). After removing the hydrogen atom above N5, the RESP charges have been recalculated using quantum calculations. Both MAO-A and MAO-B complexes with the **Cy7-MAO** ligand were embedded in a truncated octahedral box with a 10 Å padding, solvated with TIP3P water, and neutralized by chloride ions. Energy minimization was performed in three stages using the AMBER 22 package. First, the solvent molecules and counterions were minimized while restraining the solute with the IBELLY option. Next, only the hydrogen atoms of the protein were relaxed using positional restraints (500 kcal mol⁻¹ Å⁻²) applied to the heavy atoms. Finally, the entire system was fully minimized without any restraints. Each minimization was run for a maximum of 10,000 cycles, with the first 2,000 cycles using the steepest descent method followed by the conjugate gradient algorithm. The system was equilibrated in three consecutive steps: (i) an initial NVT phase (50 ps) with positional restraints (10 kcal mol⁻¹ Å⁻²) on the solute, (ii) a restrained NPT equilibration (100 ps) with a reduced restraint weight (1 kcal mol⁻¹ Å⁻²), and (iii) a final unrestrained NPT equilibration (100 ps) at 300 K and 1 atm. Molecular dynamics simulations were performed in the NPT ensemble at 300 K and 1 atm. Temperature was regulated with a Langevin thermostat (γ = 1.0 ps⁻¹) and pressure was maintained by a Monte Carlo barostat. SHAKE constraints were applied to all bonds involving hydrogen atoms, allowing a 2 fs integration time step. A total of 200 ns of production simulation of each complex was carried out.

The simulation trajectories were processed and analyzed using Visual Molecular Dynamics (VMD) software [12]. The Root Mean Square Deviation (RMSD) of the atomic positions of the protein backbone was calculated using the RMSD Trajectory tool within VMD to assess the stability of the systems throughout the simulation. Graphical plots were generated with Grace software, and molecular structures were visualized using VMD.

**Figure** **S13.** RMSD derived by 200 ns of molecular dynamics simulations of **Cy7-MAO** in complex with (a) MAO-A and (b) MAO-B.

**Figure** **S14**. Snapshots derived from 200 ns MD, detail of aromatic cage. (a) Residues of MAO-A Tyr433 and Tyr396 are represented in purple and **Cy7-MAO** in blue; (b) residues of MAO-B Tyr434 and Tyr397 are represented in purple and **Cy7-MAO** in blue.

1. *Cell Culture*

HepG2 hepatocellular carcinoma cells and SK-Mel-103 human melanoma cells were purchased from the American Type Culture Collection (ATCC). Cells were cultured in DMEM (Dulbecco's Modified Eagle Medium) supplemented with 10% FBS (Fetal bovine serum) (Sigma, F7524) and maintained in 20% O_2_ and 5% CO_2_ atmosphere at 37°C.

1. *Western blot assays*

To determine the levels of MAO enzyme in each cellular line (SK-Mel-103 and HepG2) whole-cell extracts were obtained by using lysis buffer (25 mM Tris-HCl pH 7.4, 1 mM EDTA, 1% SDS, plus protease and phosphatase inhibitors). Cell lysates were resolved in 12% SDS-PAGE gels, transferred to nitrocellulose membranes, blocked with 5% BSA, and incubated overnight with the primary antibody for MAO-A (#ab126751, Abcam) and MAO-B (#ab137778, Abcam), respectively. Besides, GAPDH (#14C10 from Cell Signalling) was used as reference protein for normalization. Then, membranes were washed and probed with the secondary antibody conjugated to horseradish peroxidase, anti-rabbit IgG peroxidase antibodyv(#A6154, Sigma) for enhanced chemiluminescence detection (Amersham Pharmacia Biotech).

1. *In vitro cytotoxicity studies*

For the *in vitro* cytotoxicity studies, HepG2 cells were seeded in a 96-well plate (7,500 cells per well). After 48 h, cells were incubated with different concentrations of **Cy7-MAO** probe (diluted in DMEM) for 48 h. The cell viability was determined by WST-1 reagent, which was added for 30 min, and then absorbance was measured at 450 nm at Wallac 1420 Victor2 Microplate Reader (Perkin Elmer).

1. *Confocal in vitro experiments*

SK-Mel-103 and HepG2 cells were seeded in a cover slip in a 6-well plate at 250,000 cells per well. After 24 h cells were incubated with **Cy7-MAO** (100 µM) for 2 h. Then cells were washed, and coverslips mounted to confocal visualization. Hoechst 33342 was added at 2 µg·mL^-1^ for nuclei staining. Confocal images were acquired in a Leica TCS SP8 AOBS confocal microscope (λ_exc_ = 552 nm; λ_em_ = 574-765 nm). Images were quantified by Image J software.

1. *Young and naturally aged BALB/cByJ mice*

BALB/cByJ mice were acquired from ENVIGO and maintained in ventilated racks under controlled temperature and pathogen-free conditions at Principe Felipe Research Centre (Valencia, Spain), provided with food and water ad libitum and alternate dark and light cycles lasting 12 h. All animals were treated humanely, and experiments were approved by the Ethical Committee for Research and Animal Welfare Generalitat Valenciana, Conselleria d`Agricultura, Medi ambient, Canvi clim`atic i Desenvolupament Rural (2023-VSC-PEA-115).

A first evaluation was performed using female and male BALB/cByJ mice of 2-month-old (n = 5) and 12-month-old (n = 5) female and male BALB/cByJ mice to validate Cy7-MAO in young and aged groups, respectively. Based on these results, a second set of experiments was conducted using female and male BALB/cByJ mice of various ages—2 months (n = 3), 5 months (n = 3), 8 months (n = 3), and 14 months (n = 2)—to explore the correlation between urinary fluorescence and age-related MAO activity. In both cases, the experimental procedure was as follows:

Mice were anesthetized by inhalation of 2% isoflurane and intraperitoneally injected with **Cy7-MAO** (10 mM, 200 µL) in PBS. Fluorescence was monitored in an IVIS spectrum (Perkin Elmer) for 15 min (λ_exc_ = 535 nm; λ_em_ = 640 nm; time exposure: 10 s). Urine was collected after mice recovered from anaesthesia in an Eppendorf tube and analysed directly by IVIS (λ_exc_ = 535 nm; λ_em_ = 640 nm; time exposure: 1s). Finally, mice were euthanized, and organs were harvested for further MAO expression characterization.

Fluorescence measurements of collected urine samples were also analyzed with a fluorescence spectrophotometer (JASCO FP-8500). For this purpose, 5 µL of urine were diluted in 95 µL of distilled water and fluorescence spectra were recorded at 640 nm (λ_exc_ = 535 nm). The amount of Cy7 fluorophore excreted in urine was calculated through a calibration curve. For the calibration curve, a stock solution of Cy7 in blank urine from an untreated young mouse was prepared. Serial dilutions were prepared in the same urine and 5 µL of each Cy7 urine solution was added to 95 µL of distilled water and measured in the fluorimeter under the same condition.

HPLC-MS measurements were obtained using an eluent gradient method from H_2_O-acetonitrile (100:0 v/v) to H_2_O-acetonitrile (0:100 v/v) at 20 min with a flow rate of 0.4 mL·min^-1^ with a kromasil C18 column. Mass spectroscopy chromatograms were recorded with an Agilent Ultivo mass spectrometer equipped with a triple Q-TOF detector using a dual selected ion monitoring (SIM) function at 745 m/z and 705 m/z simultaneously, corresponding to the **Cy7-MAO** and Cy7 fluorophore, respectively. The calibration curve was obtained by measuring known concentrations of the **Cy7-MAO** probe and the fluorophore in H_2_O using the dual SIM method. The amount of **Cy7-MAO** and Cy7 fluorophore excreted was calculated by measuring 5 µL of urine sample diluted in 95 µL H_2_O and multiplying the corresponding concentration, obtained with the calibration curve, by the volume of urine collected for each sample. The percentage of probe or dye excreted in the urine was obtained by relating it to the amount of **Cy7-MAO** probe injected.


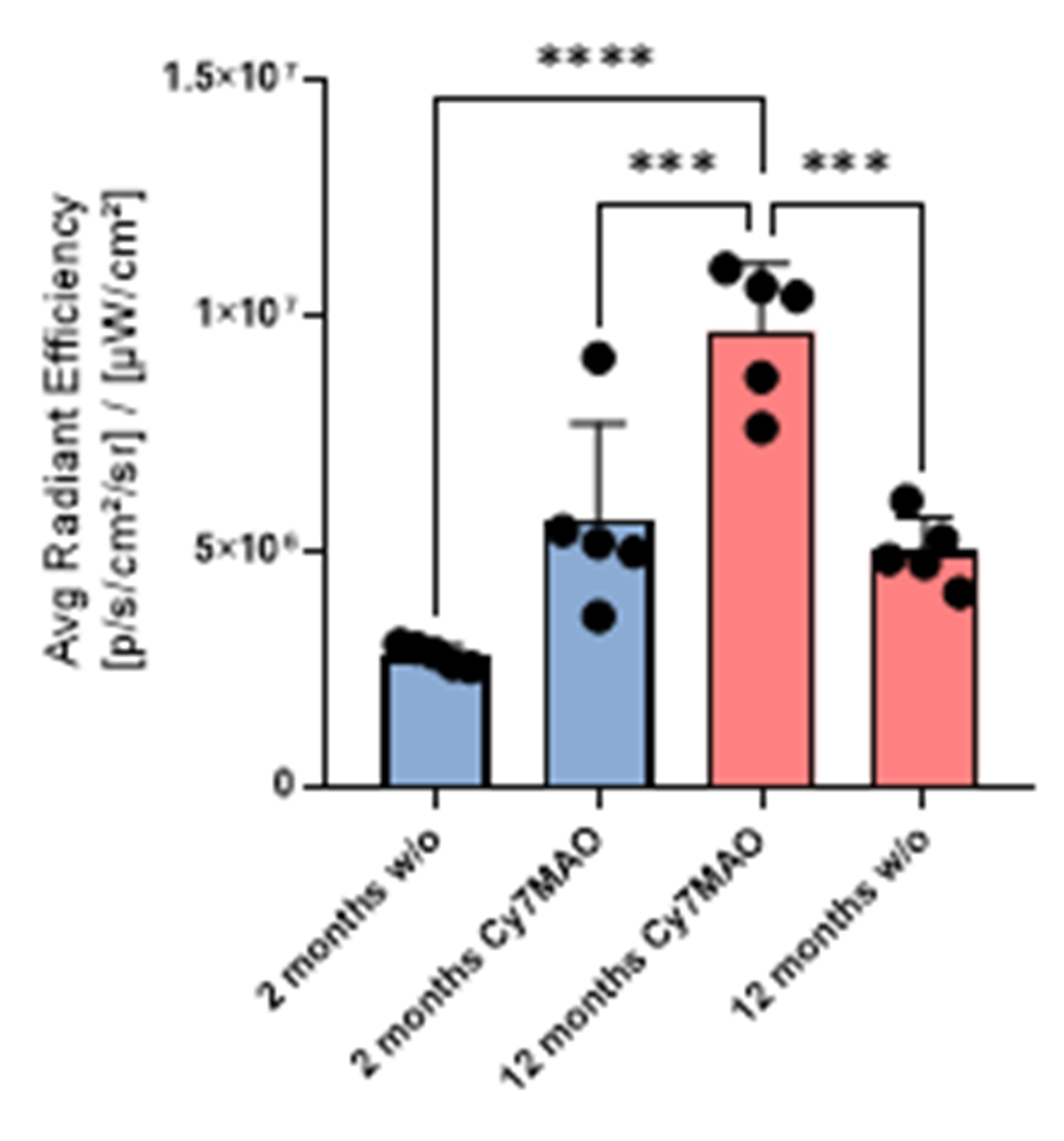


**Figure S15.** Quantification of IVIS images of bladders. Values are expressed as mean ± SD. Statistical analysis was assessed by applying One-Way ANOVA with multiple comparisons (****p <0.0001, ***p < 0.001).

1. *MAO expression by RT-qPCR*

The frozen organ samples (brain, lungs, liver and kidneys) were employed for total RNA extraction. The tissue was homogenized and RNA isolated with TRIzol according to the manufacturer's instructions (Merck, T9424). The total RNA was quantified using NanoDrop™. Samples were treated with *DNase I* (Nzytech, MB19901) to avoid genomic DNA contamination. The retrotranscription reaction of total RNA was performed using the PrimeScript^TM^ RT Reagent Kit (Takara, RR037A) following the manufacturer’s protocol. Quantitative PCR reactions were performed using qPCR Green Master Mix (2x) (Nzytech, MB22402) with each sample loaded in triplicate and were run in a LightCycler® 480 System (Roche). Data were analyzed using the LightCycler 480 relative quantification software. Nucleotide sequences of the primers used for mRNA expression analyses are listed in Table S2.

**Table S2**. Oligonucleotide sequences used for gene expression analysis by RT-qPCR.

| **Gene** | **Primer** | **Sequence (5’→3’)** |
| --- | --- | --- |
| *Maoa* | Forward | AACTTACCCATTCCGTGGTG |
|  | Reverse | CCACAGGGCAGATACCTCAT |
| *Maob* | Forward | CCTTGCTGAAGAGTGGGACT |
|  | Reverse | TGTCCTCCATTGGTTGTTGA |
| *Actb* | Forward | GTCCACACCCGCCACC |
|  | Reverse | ACCCATTCCCACCATCACAC |

**Figure S16.** (a) mRNA expression levels of *Maoa* and (b) Maob in major organs corroborated by real-time PCR. The expression of significantly changed in old mice versus young mice. *Actb* was used for input normalization. Values are relative to control mice and are expressed as mean ± SD. Statistical significance was assessed by the two-tailed Student’s *t*-test: *p < 0.05; **p < 0.01; **p < 0.001; ****p < 0.0001 (n=5).

1. *Biocompatibility studies*

BALB/cByJ mice were intraperitoneally injected with **Cy7-MAO** (10 mM, 200 µL) or PBS (n=6 animals per group), and body weight was monitored for 2 weeks. Intracardial blood was collected with a heparinized syringe for hematological analysis, and mice were euthanized by cervical dislocation. Major organs were fixed in 4% paraformaldehyde and embedded in paraffin for hematoxylin-eosin staining.


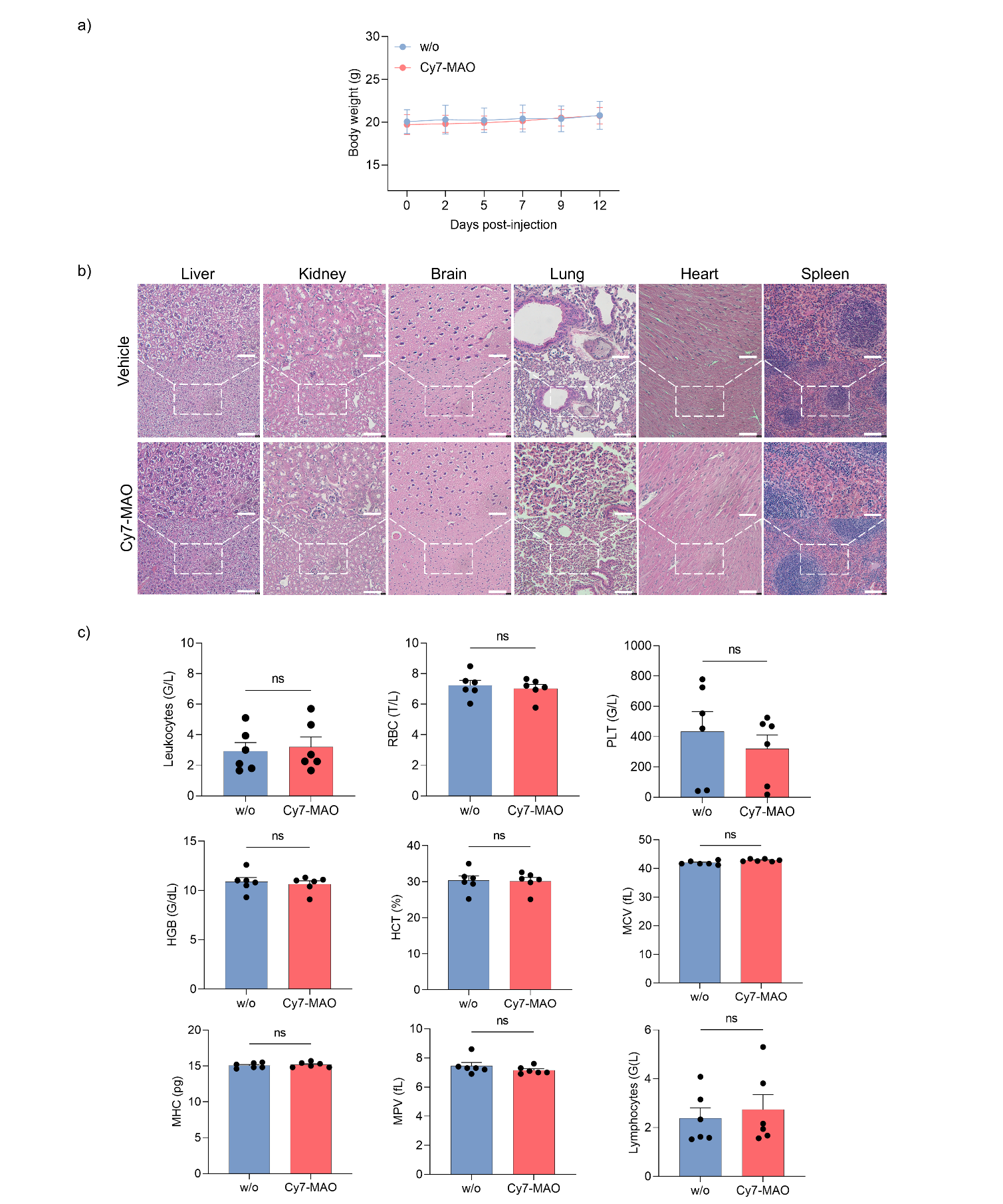


**Figure S17.** (a) Change in body weight of mice after injection with **Cy7-MAO** (10 mM) or PBS and up to endpoint. Data presented as mean ± SD (n = 6 per group) (b) Hematoxylin and eosin staining of major organs (liver, kidney, brain, lung, heart and spleen). Scale bar: 100 µm; enlargements: 25 µm. (c) Hemogram analysis of each group at endpoint, including leukocytes, red blood cells (RBC), platelets (PLT), hemoglobin (HGB), hematocrit (HCT), mean corpuscular volume (MCV), mean corpuscular-hemoglobin (MCH), mean platelet volume (MPV) and lymphocytes. Data presented as mean ± SEM, n =6 animals. Statistical differences were assessed by applying unpaired *t*-test.

1. *Validation in additional mouse strains of different ages*

SAMP8 mice and C57BL/6 mice were housed at the Universitat de València (UV). All mice were bred and housed, under 12 h periods of light/darkness, at a temperature of 20-22 °C with 40-60% humidity, and a freely accessible diet of pellets and water following European Union 2010/63/UE and Spanish RD-53/2013 guidelines and under official veterinary supervision. All animals were treated humanely, and experiments were approved by the Ethical Committee for Research and Animal Welfare Generalitat Valenciana, Conselleria d`Agricultura, Medi ambient, Canvi climàtic i Desenvolupament Rural (2019-VSC-PEA-0284) and conducted them following the recommendations of the Federation of European Laboratory Animal Science Associations (FELASA).

SAMP8 of 2-months (n = 5) and 8-months (n=5) and C57BL/6 of 2-months (n=5) and 16-months (n = 5) were anesthetized by inhalation of 2% isoflurane and intraperitoneally injected with Cy7-MAO (10 mM, 100 µL) in PBS. Urine was collected after mice recovered from anaesthesia in an Eppendorf tube, and fluorescence measurements of collected urine samples were also analyzed with a fluorescence spectrophotometer (JASCO FP-8500). For this purpose, 5 µL of urine were diluted in 95 µL of distilled water and fluorescence spectra were recorded at 640 nm (λ_exc_ = 535 nm). The amount of Cy7 fluorophore excreted in urine was calculated through a calibration curve. For the calibration curve, a stock solution of Cy7 in blank urine from an untreated young mouse was prepared. Serial dilutions were prepared in the same urine and 5 µL of each Cy7 urine solution was added to 95 µL of distilled water and measured in the fluorimeter under the same condition.

1. Rojas-Vázquez S, Lozano-Torres B, García-Fernández A, Galiana I, Pérez-Villalba A, Martí-Rodrigo P, *et al*, Martínez-Máñez, R, *Nat. Commun.* **2024**, *15*, 775.
2. Bondar K, Bokan M, Gellerman G, Patsenker L D, *Dyes Pigm*. **2020**, *172*, 107801.
3. Shrivastava A, Gupta V B. Methods for the determination of limit of detection and limit of quantitation of the analytical methods. *Chron. Young Sci.* **2011**, *2*, 21-25.
4. Frisch M J, *et al*. Gaussian 16, Revision A.03, Gaussian, Inc., Wallingford CT, **2016**.
5. Son S, Ma J, Kondou Y, Yoshimura M, Yamashita E, Tsukihara T, Proc. Natl. Acad. Sci. U.S.A. **2008**, 105, 5739-5744.
6. Iacovino L G, Reis J, Mai A, Binda C, Mattevi A, *ChemMedChem.* **2020**, *15*, 1394-1397.
7. Morris G M, Goodsell D S, Huey R, Hart W E, Halliday S, Belew R, Olson A J. AutoDock. automated docking of flexible ligands to receptor-User Guide. **2021**
8. Case D A, *et al.*, Amber 2022, University of California, San Francisco, **2022**.
9. Chuan T, Kasavajhala K, Belfon K A A, Raguette L, Huang H, Migues, A N, Bickel J, Wang Y, Pincay J, Wu Q, Simmerling C, *J*. *Chem*. *Theory* *Comput*. **2020**, *16*, 528-552.
10. Wang J, Wolf R M, Caldwell J W, Kollman P A, Case D A, J. Comput. Chem. 2004, 25, 1157-1174.
11. Humphrey W, Dalke A, Schulten K, *J*. *Mol*. *Graph*. **1996**, *14*, 33-38.
12. Antony J, Medvedev D M, Stuchebrukhov A A, *J*. *Am*. *Chem*. *Soc*. **2000**, *122*, 1057-1065.
